# A Comprehensive Review: Current Strategies for Detoxification of Deoxynivalenol in Feedstuffs for Pigs

**DOI:** 10.3390/ani15182739

**Published:** 2025-09-19

**Authors:** Changning Yu, Peter Plaizier, Joshua Gong, Chengbo Yang, Song Liu

**Affiliations:** 1 Department of Biosystems Engineering, University of Manitoba, Winnipeg, MB R3T 2N2, Canada; changni1@ualberta.ca; 2 Department of Animal Science, University of Manitoba, Winnipeg, MB R3T 2N2, Canada; pplaizier367@gmail.com (P.P.); joshua.gong@agr.gc.ca (J.G.); 3 Guelph Research and Development Centre, Agriculture Agri-Food Canada, Guelph, ON N1G 5C9, Canada

**Keywords:** deoxynivalenol, detoxification, strategies, feed, pigs

## Abstract

Deoxynivalenol (DON) is a mycotoxin produced by *Fusarium graminearum* and *Fusarium culmorum*, primarily contaminating wheat, corn, and their derivatives. Although less toxic than some other mycotoxins, DON is significant due to its abundance, particularly in wheat, and its resistance to in vitro detoxification. Compared to other livestock, pigs are the most susceptible animals to DON’s presence due to its interaction with wheat-heavy diets. The best way to attenuate the effects of DON is to prevent its presence on cultivated land; however, given that it is difficult to prevent the occurrence of DON in freshly harvested cereals completely, other strategies must be applied. For pigs, chronic consumption of feed with DON concentrations below even the regulated limits can still negatively impact growth. Thus, DON-detoxifying agents including antidotes, DON binding agents, and exogenous in vivo enzyme additives, have been added to feed in an attempt to detoxify DON. This comprehensive review highlights the most recently developed DON-detoxification techniques and assesses each treatment’s viability and efficacy, with an emphasis on chemical and biochemical techniques and the rapid development of artificial intelligence (AI) technologies. Herein we explore the unmet needs and future directions of current technologies while discussing promising strategies that can advance the DON-detoxification field.

## Simple Summary

With the ongoing progression of global climate change, the prevalence of grain contamination by mycotoxins has been steadily rising. Among these, deoxynivalenol (DON, commonly known as vomitoxin) presents significant challenges for complete detoxification in vitro without adversely affecting the nutritional quality of grains. In response, numerous strategies have been investigated to achieve effective detoxification of DON both in vitro and in vivo. Concurrently, the rapid development of artificial intelligence (AI) technologies has shown promise in facilitating efficient DON detoxification processes. This review provides a comprehensive review of current DON detoxification strategies and examines the emerging role of AI in this field, with the aim of supporting the development of effective grain detoxification strategies in the pig industry.

## 1. Introduction

Mycotoxin refers to a diverse array of chemical compounds that are produced as secondary metabolites by various fungi genera, including *Aspergillus*, *Penicillium*, and *Fusarium*, through natural processes [1,2]. More than 500 mycotoxins have been identified and explored in scientific studies [3,4]. Around 25% of agricultural products worldwide are estimated to be contaminated with mycotoxins each year, leading to economic losses, and causing a spectrum of toxic effects in humans and animals [5]. With the evolving global climate, the prevalence of mycotoxin-contaminated crops is expected to increase [6]. Numerous studies have documented mycotoxin-induced toxic effects in humans, encompassing conditions such as necrosis, hepatitis, hemorrhage, gynecological inflammation with testicular atrophy, neurological disorders, cancer, and, in severe cases, fatality [7,8]. In animals, mycotoxin exposure can lead to decreased feed intake, chronic illnesses, deteriorating health, potential mortality, and lowered reproductive output [9,10]. Despite rigorous preventative strategies in the agricultural sector, mycotoxin contamination remains a pervasive challenge across the entire food production and storage chain, posing a significant threat to human and animal health and well-being. Furthermore, the annual economic impact associated with mycotoxin contamination in food and feed is escalating, with estimated losses reaching billions of dollars each year in North America alone [11].

**Figure 1:**
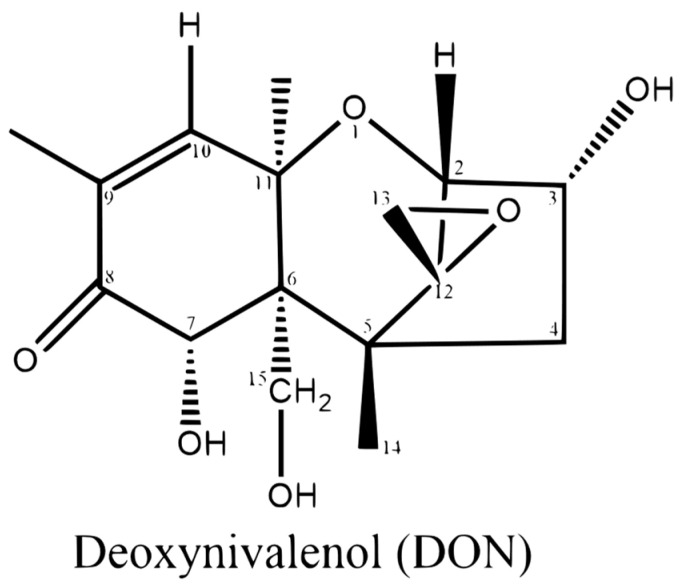
The molecular structure of deoxynivalenol (DON).

Within the feed industry, prevalent mycotoxins encompass deoxynivalenol (DON), zearalenone (ZEA), aflatoxin (AFLA), T-2 toxin (T-2), ochratoxin (OTA), HT-2 toxin (HT-2), and fumonisin (FUM). It is noteworthy that DON has emerged as the primary non-nutritional factor influencing feed value due to its widespread occurrence, challenges in detoxification in vitro and in vivo, and adverse effects on human and animal health, alongside economic repercussions for producers and consumers. Hence, gaining a deeper understanding of DON toxicity and properties necessitates comprehending its background and molecular structure. DON was initially identified and designated after its isolation from *Fusarium*-infected barley in Japan [12,13]. Simultaneously, researchers in the United States independently detected the same compound in *Fusarium*-infected corn and labeled it “vomitoxin” due to its propensity to induce vomiting in pigs [14]. Structurally, DON is a polar organic compound with a double bond at C9-C10, a free hydroxyl group, and an epoxide group at C12-C13, which plays a critical role in its toxicity (Figure 1). The C12-C13 epoxide group of DON can react with ribosomes, leading to the ribotoxic stress response, which activates various protein kinases, hindering the modulation of gene expression, and suppressing protein synthesis [15]. Recent studies have also discovered that the C-3 hydroxyl group in DON contributed to the toxicity of the compound; thus, conversion to 3-epi-DON or 3-keto-DON could be a strategy to detoxify DON [16,17]. Although DON exhibits lower toxicity compared to other mycotoxins like ZEA, T-2, FUM, OTA, and AFLA, two noteworthy characteristics that differentiate DON from other mycotoxins and raise concerns are its prevalence and propensity to contaminate cereals commonly utilized for food and feed along with the absence of straightforward in vitro detoxification methods [18,19]. One crucial physicochemical characteristic of DON is its capacity to endure elevated temperatures, posing a risk of its presence in feed/food even after processing. A previous study revealed that 1.75–2.25 mg/kg DON in contaminated food remained unchanged after processing at temperatures ranging from 100 °C to 120 °C and a pH of 4.0 to 7.0 for 60 min; however, entire degradation of DON occurred with a 30 min treatment at 120 °C or a 15 min treatment at 170 °C, provided that the pH was adjusted to 10 [20]. Meanwhile, frying the 1.2 mg/kg DON-contaminated food in oil did not decrease concentrations efficiently, which reduced to 28% at 169 °C for 15 min, 21% at 205 °C for 2.5 min, and 20% at 243 °C for 1 min [18,21]. The heat resistance of DON presents a significant challenge, making the removal of DON from contaminated grains through thermal treatment difficult. In addition, the uncertainty surrounding DON’s potential thermal decomposition products and the absence of toxicity studies on these by-products cast doubt on the viability of employing high temperatures or heating degradation methods for DON [22].

Concerns emerge regarding the contamination of grains by DON in the North American region. A recent Biomin 2023 survey in North America revealed that DON prevalence in 991 cereal samples surpassed that of all other mycotoxins, reaching 79%, exceeding the 76% recorded in the 2022 survey [23]. Additionally, within the Canadian Grain Commission harvest sample program, DON was identified in 75% of the 54 samples, with a maximum concentration of 4.7 mg/kg [24]. The data presented above showed the importance of addressing DON contamination in cereals through heightened attention and effective interventions.

Due to increasing awareness of food safety, many countries have established limits for DON concentrations in animal feed. For instance, in the European Union (EU), the maximum level of DON in the feedstuffs for pigs is 0.9 mg/kg, while the US Food and Drug Administration (FDA) and Agriculture and Agri-Food Canada (AAFC) recommend that the maximum concentration of DON in feed for pigs should not exceed 1.0 mg/kg [25,26,27]. Typically, two prevalent strategies are employed to alleviate the adverse impacts of DON: controlling plant pathogens (*Fusarium* spp.) to reduce DON production and detoxifying DON already existing in infected cereals through both in vitro and in vivo approaches. Several methods, including pre-harvest control, harvesting control, and post-harvest control, have been attempted to control the occurrence of this toxin in plants. However, even the use of best practice agricultural methods, such as crop variety selection, sowing date, suitable cultivation techniques, predictive models, avoiding insect attack, and biocontrol techniques, might be insufficient in preventing the production of DON after the harvest when the raw products were stored in environments suitable for the generation of DON [28,29]. Hence, there is an ongoing necessity to implement post-harvest detoxification methods for DON, employing physical, chemical, biological, and nutritional approaches.

Pigs, especially piglets, exhibit heightened sensitivity to DON cytotoxicity among various livestock species, attributed to rapid absorption of DON in the porcine intestine, limited biotransformation of DON to the non-toxic form de-epoxidation DON (DOM-1) by intestinal microorganisms, and a high proportion of wheat or corn in their diets [30]. Studies have demonstrated that DON levels ranging from 1.0 to 4.0 mg/kg in pig diets resulted in decreased feed intake and weight gain due to feed refusal, potentially linked to a reduction in nutritional value [31,32]. When DON reached 20 mg/kg in pigs’ diets, it induced emesis, leukocytosis, intestinal hemorrhaging, and, in severe cases, circulatory shock [33]. Investigations into the effects of various sources and levels of DON on diverse stages of pigs were extensively documented, as detailed in Table 1.

This review aims to summarize the metabolism of DON in pigs, evaluate the latest DON detoxification strategies, and assess the viability and efficacy of these approaches, with particular focus on chemical and biochemical methods. Additionally, it examines the existing gaps and future directions of current technologies, highlighting innovative strategies that may advance the field of DON detoxification. Furthermore, the potential application of artificial intelligence (AI) techniques in the future detoxification of DON in feed is explored.

## 2. Materials and Methods

In this review, information from journal articles, books, reports, and regulations was thoroughly collected and analyzed. Literature searches were conducted using the databases PubMed, Web of Science, ScienceDirect, Google Scholar, and Scopus with the following keywords and search strings: “the metabolism of DON in pigs,” “DON in pigs,” “occurrence and prevention of DON,” “physical detoxification of DON,” “chemical detoxification of DON,” “biological detoxification of DON,” “nutritional strategies in DON detoxification in pigs,” and “artificial intelligence (AI) in DON detoxification.” The last database access was in June 2025. Titles and abstracts of the retrieved articles were initially screened against the following inclusion criteria: (1) Studies evaluating the effects of different levels of DON in pigs, with emphasis on DON toxicity, absorption, distribution, metabolism, excretion, and DON-mediated signal transduction. (2) Studies describing the occurrence and prevention of DON, including identification of environmental conditions that favor its production. (3) Research on physical strategies for DON detoxification such as sorting, washing, dehulling, heating or thermal processing, milling, extrusion, and irradiation. (4) Studies evaluating chemical strategies for DON detoxification, including alkalization, reduction, hydrolysis, ammoniation, and oxidation, with ozone and organic acids also considered. (5) Studies addressing biological strategies for DON detoxification and their underlying mechanisms. (6) Studies investigating nutritional strategies for DON detoxification under different levels of contamination. (7) Research proposing future perspectives on the application of artificial intelligence (AI) to identify and predict DON-degrading enzymes, thereby advancing detoxification approaches.

The search results were refined, and the relevant information was analyzed, categorized, and organized into sections to comprehensively address the scope of this comprehensive review.

## 3. Deoxynivalenol (DON) in Pigs: Toxicity and Ingestion Fate

### 3.1. DON Toxicity in Pigs

**Figure 2:**
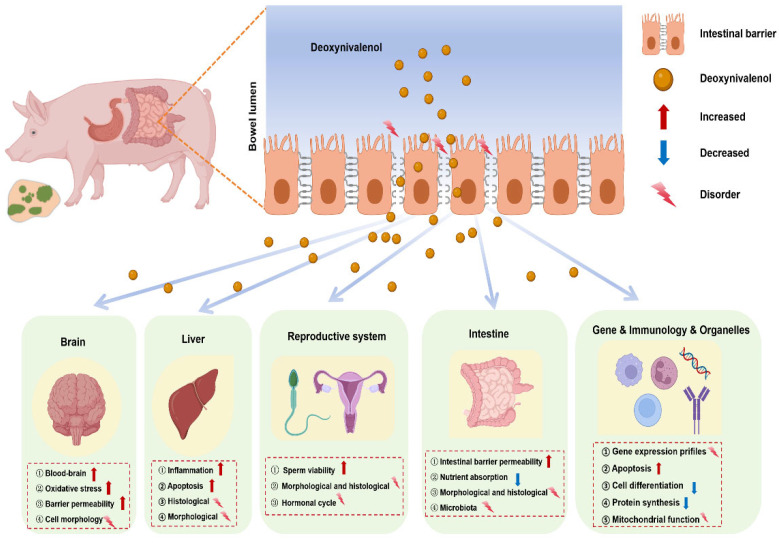
The impacts of deoxynivalenol (DON) on the organs and systems of pigs.

The consumption of DON by pigs results in both acute and chronic toxicity, including cytotoxicity, immunotoxicity, neurotoxicity, reproductive toxicity, and the risk of carcinogenesis, teratogenesis, and mutagenesis. Figure 2 provides a summary of the impact of DON on the organs and systems of pigs. As mentioned earlier, the heightened susceptibility of pigs to DON-induced toxicity was primarily attributed to the rapid absorption of the toxin in the upper digestive system, particularly in the small intestine [51]. Consequently, the effects of DON on pigs have been a focal point of interest and extensive study. Furthermore, current research showed that exposure to DON, even below the regulatory concentration limits, could also produce deleterious effects on pigs [45]. Prior to identifying and critically analyzing DON detoxification strategies, it is imperative to understand the mechanisms in which it affects pigs. Therefore, discussing the absorption, distribution, metabolism, excretion, and signal transduction mediated by DON in pigs is necessary.

### 3.2. The Absorption of DON in Pigs

Swine demonstrates a significantly higher absorption rate of ingested DON than other livestock species. Prior studies have demonstrated that swine rapidly absorbed up to 82% of orally administered DON, whereas, in cows, less than 1% of the administered dose was systemically absorbed [52,53]. In pigs, the proximal part of the small intestine is the primary site of DON absorption, especially jejunal tissues. DON is excreted through the urinary and biliary pathways, but urinary elimination is the main route without any accumulation in tissues [54]. Upon oral administration of DON to pigs, rapid absorption occurred, resulting in detectable concentrations within 0.11–1.32 h in serum and time of maximum serum DON concentrations (T_max_) values ranging from 0.2 to 4.71 h [53,55,56,57,58]. The prompt detection of DON in serum signified the rapid absorption of DON and suggested that this absorption might initiate in the upper portion of the duodenum [51]. Multiple pivotal factors, such as species, age, gender, and the source and dosage of DON, would influence the absorption rate of DON in pigs.

### 3.3. The Distribution of DON in Pigs

Studies using radio-labeled DON found that DON and its metabolites were transiently distributed in all tissues of treated pigs and chickens [53,59]. The rapid distribution of DON throughout tissues might contribute to the short half-life observed in swine, with one study finding DON levels in plasma peaked within 15–30 min after intragastric dosing, with a half-life of 3.9 h [60]. Pure DON and DON-contaminated feed, as distinct sources of DON, resulted in varying peak plasma times in pigs. Prelusky et al. [61] observed that peak concentrations of DON in pig serum, reaching 367 µg/L, occurred at 3.75 h following the administration of an intragastric dose of 1 mg/kg pure DON, with around half of the systemically absorbed DON being excreted within 5.9 h. Dänicke et al. [51] demonstrated that the peak serum concentration of DON (7.67 ng/mL) occurred at 4.1 h after pigs ingesting DON-contaminated feed (4.2 mg/kg), with half of the systemically absorbed DON being excreted within 5.8 h. The variation in their findings might be attributed to the disparate levels and sources of DON. Additionally, DON passage has been shown to cross the pig placenta [62]. A previous study revealed the presence of DON in fetal plasma, liver, and kidneys when pregnant sows were exposed to DON-contaminated diets, and the exposure of pregnant sows to DON was associated with fetal growth restriction [63]. This indicated that the consumption of DON by sows could influence the health of their offspring through the maternal transmission of the toxin.

### 3.4. The Metabolism and Excretion of DON in Pigs

One prior study has illustrated that DON was rapidly and effectively absorbed but poorly metabolized in pigs, contributing to their heightened sensitivity to DON toxicity [55]. After ingestion in pigs, DON undergoes intestinal and hepatic metabolism, forming its derivative compounds such as DON-3-glucuronic acid (DON-3-GlcA, D3G), DON-15-glucuronic acid (DON-15-GlcA, D15G), and DOM-1. D3G and D15G emerged as the principal metabolites, together comprising around 40–50% of the DON excreted in the urine, while the remaining portion primarily consisted of free DON [64,65]. Upon analysis of urine samples from pigs, it was observed that concentrations of D3G and D15G were comparable, albeit with slightly higher concentrations of D15G detected in the latter [66]. Pierron et al. [67] illustrated through experimentation utilizing Caco-2 human cells that the IC_50_ value of D3G was 10 µM, which was notably 7.7 times greater than that of DON, estimated at 1.30 µM under identical experimental conditions. Broekaert et al. [68] reported comparable findings, indicating that D3G displayed markedly reduced cytotoxic effects on IPEC-J2 cells relative to DON; however, the calculation of the IC_50_ for D3G was precluded due to the observation that cell viability upon exposure to D3G seemed to be unaffected by varying concentrations.

In pigs, the excretion of DON and its metabolites occurs predominantly through urine. Prelusky et al. [53] reported that more than 86% of orally administered DON was excreted through urine in swine. Additional studies corroborated this assertion by demonstrating a higher percentage of DON excretion in urine (ranging from 39 to 90%) compared to feces [51,55,69]. Likewise, DON’s derivatives are also mainly excreted through urine in pigs. For instance, Eriksen et al. [69] found that pigs fed a diet containing 2.5 mg/kg of 3-Acetyl-DON (3-ADON) exhibited a substantial excretion of free DON (45%) and glucuronide-conjugated DON (42%) in urine (87%), with a minimal fraction of 3-ADON excreted in feces (2%), and de-epoxidized DON representing 52% of fecal metabolites for 3-ADON. The results mentioned above showed that 86% of DON was excreted in urine, and it seemed that only the remaining 14% of DON exerted toxic adverse effects on pig health [53]. Although excretion values suggest a low impact of DON levels on pigs, it is imperative to consider that the DON acetyl-derivatives such as 3-ADON and 15-acetyl-DON (15-ADON) also possess the potential to affect pig health adversely. Like DON in pigs, one previous study found that 10 μM of 3-ADON and 15-ADON could also change the intestinal barrier function of IPEC-1 cells by altering the gene expressions of tight junction proteins [70].

### 3.5. Signal Transduction Mediated by DON in Pigs

The intestinal tract, acting as the initial defense against food contaminants, demonstrated heightened sensitivity to DON and other mycotoxins [71]. Following exposure to DON, pigs primarily absorb it in the upper segment of the small intestine, particularly within the jejunal epithelial cells. DON primarily initiates oxidative stress, disrupts epithelial tight junctions, and provokes dysfunction in the intestinal barrier [72]. Simultaneously, it elicits proinflammatory responses and apoptosis in intestinal epithelial cells (IECs), ultimately contributing to the development of enteropathy [73,74]. This section will explore the mechanisms of DON-induced apoptosis in IECs and delve into the associated molecular signaling pathways.

The C12-C13 epoxide group of DON can bind to the 60S ribosomal subunit, thus rapidly activating mitogen-activated protein kinases (MAPK) and inducing apoptosis in a process known as the “Ribotoxic stress response” [15]. The MAPK pathway plays a pivotal role in controlling normal cell growth, differentiation, and apoptosis and is critical for signal transduction in the immune response [75,76]. It is reported that DON can cause the up-regulation of MAPK-mediated expression of proinflammatory cytokines, chemokines, and apoptosis [73]. DON has been shown to activate MAPK in the intestines of pigs [77]. The upstream families of MAPK include kinases, hematopoietic cell kinase (Hck), and double-stranded RNA-(dsRNA)-activated protein kinase (PKR) [78]. Hck is a member of the Src non-receptor tyrosine kinases, and a distinct family of protein tyrosine kinases called the “rheostats for immune signaling” [79]. PKR, a widely expressed serine/threonine protein kinase, can be activated by dsRNA, interferon, and other agents [80]. Meanwhile, the downstream families of MAPK comprise a family of serine and threonine kinases, including extracellular signal-regulated kinase (ERK), Jun N-terminal kinase (JNK), and p38 [81]. JNK and ERK are involved in regulating both cell survival and death depending on cell types and stimulus, whereas p38 can promote apoptosis via p53 activation [82]. ERK 1/2 is of particular importance because it can be involved in intestinal epithelial cell morphology and in the structure of tight junctions that regulate the barrier function of the intestinal tract [73]. Previous studies have identified that DON could activate ERK, JNK, and p38 in vitro and in vivo, which indicated that the ribotoxic stress response might mediate DON toxicity [83,84]. In in vivo and ex vivo models, Lucioli et al. [77] illustrated that fed diets containing 2.3 mg/kg of DON to pigs for 35 days and exposed to 10 µM DON in jejunal explants for 4 h could upregulate the intestinal expression of phosphorylated MAPK, ERK 1/2, and p38, without increasing the phosphorylation of JNK, consequently resulting in intestinal morphological changes, such as apical lysis of enterocytes and villi atrophy. Zhang et al. [73] obtained similar results and found that 1.0–2.0 μg/mL DON efficiently elevated the phosphorylation of p38, ERK1/2, and JNK in IPEC-J2 cells, leading to the proinflammatory cytokines production, such as IL1A, IL6, and TNF-α. However, Wang et al. [85] illustrated that 1000 ng/mL DON activated the mRNA and protein expression of JNK and p38 while inhibiting the activation of ERK, ultimately resulting in cell apoptosis in piglet hippocampal nerve cells (PHNCs). Their different results might be due to different cell models, but the intestinal toxicity of DON certainly involved MAPK activation in pigs.

Apoptosis plays a crucial role in diverse processes, encompassing normal cell turnover, the development and functioning of the immune system, hormone-dependent atrophy, embryonic development, and chemically induced cell death [86]. Autophagy exhibits dual roles, encompassing both anti-stress mechanisms and physiological functions. Specifically, autophagy facilitates the generation of new building blocks and energy for anabolism during nutrient deprivation or stress through the degradation of intracellular components, while also contributing to cell homeostasis regulation and disease prevention under normal conditions by eliminating damaged organelles or harmful components [87,88]. Apoptosis and autophagy are essential for preserving internal environment homeostasis and promoting healthy growth [89]. Several vital points characterize the regulation of DON-induced cell apoptosis within the mitochondria-initiated pathway: the functional implications of mitochondria are integral, with noticeable detection of abnormal structural alterations; cytochrome c release from the mitochondria triggers caspase activation of caspase-9, which subsequently cleaves and activates downstream caspase-3; permeabilization of the mitochondrial outer membrane, initiated by alterations in Bcl-2 family expression, leads to the release of intermembrane space proteins into the cytosol [90]. Treatment with DON disrupts the balance between pro-apoptotic and anti-apoptotic factors, thereby inducing apoptosis. Mitochondria serve as the cellular “powerhouses” by functioning as metabolic centers and signaling platforms crucial for providing energy [72]. DON disrupts the functioning of mitochondria by inducing the opening of the mitochondrial permeability transition pore, leading to the dissipation of mitochondrial membrane potential, which results in an imbalance in mitochondrial fission/fusion dynamics and triggers mitophagy within intestinal cells [91]. DON modifies the normal function of mitochondria and releases free radicals, including reactive oxygen species (ROS), and reduces the antioxidant activity of enzymes such as total superoxide dismutase (T-SOD), catalase (CAT), and *glutathione* peroxidase (GSH-Px) within the cell, leading to an increase in lipid peroxidation, altering cell membrane integrity [92]. The primary factor contributing to cellular oxidative stress is the heightened production of ROS coupled with a reduction in cellular antioxidant capacity [93]. Elevated ROS production and lipid peroxides are also associated with lipids, proteins, and DNA damage [94]. Kang et al. [95] found that DON concentrations ranging from 0.5 to 6.0 µg/mL adversely impacted the concentration and activity of CAT and GSH-Px, elevated ROS production, inducing oxidative stress, cell apoptosis, and inflammatory response in IPEC-J2 cells. Ren et al. [96] also reported that DON concentrations ranging from 0.1025–0.82 µg/mL significantly elevated levels of ROS and malondialdehyde (MDA) while concurrently reducing the activities of total antioxidant capacity (T-AOC), succinate dehydrogenase (SDH), and SOD, resulting in oxidative damage, ROS generation, inhibition of mitochondrial fusion, and promotion of mitochondrial autophagy. Hence, DON can cause intestinal epithelial cell damage by inhibiting mitochondrial function and producing large amounts of ROS. Additionally, previous studies revealed the regulation of autophagy and apoptosis through the modulation of p38, JNK, PI3K-AKT-mTOR, JAK2/STAT3, and AMPK signaling pathways [74,97,98,99,100]. The above findings can contribute to a novel understanding of the molecular mechanisms underlying the cytotoxic effects of DON incurred.

**Figure 3:**
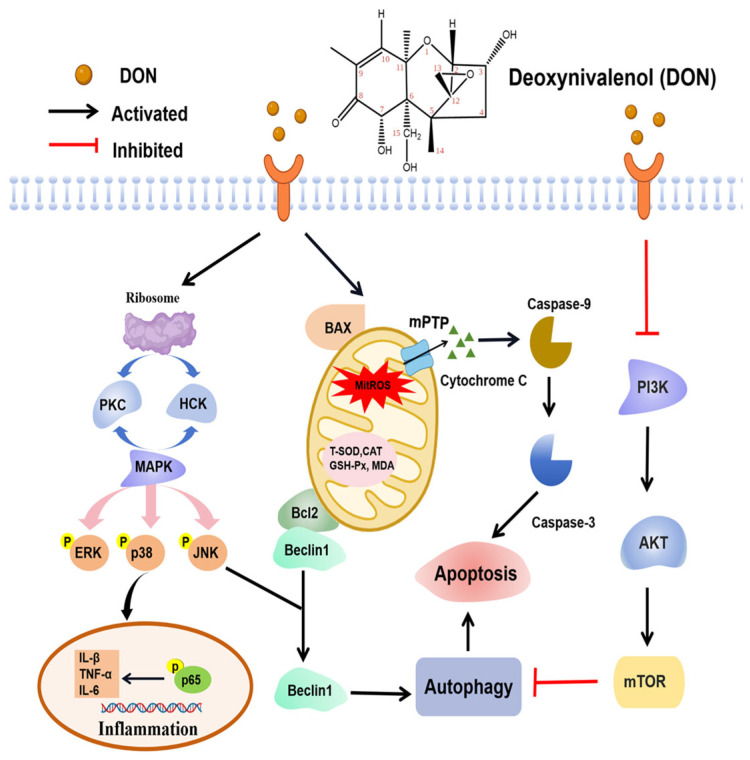
Signal transduction is mediated by deoxynivalenol (DON) in the process of apoptosis. DON triggers the activation of the mitogen-activated protein kinase (MAPK) pathway, leading to DNA fragmentation and halting the progression of the cell cycle in mammalian cells. Simultaneously, it prompts swift p38 phosphorylation near the ribosome, stimulates changes in mitochondrial Ca^2+^ concentrations, and modulates the expression of various proinflammatory cytokines.

In short, MAPK is a critical signaling pathway for the toxic mechanism of DON. PKR and Hck have been identified as key upstream kinases that trigger the downstream events induced by DON. Besides PKR and Hck, the accumulation of ROS by DON can also induce cell apoptosis and autophagy. Furthermore, in pigs, DON may induce cytotoxicity and apoptosis by activating autophagy by suppressing the PI3K-AKT-mTOR signaling pathway and activating the JAK/STAT3 signaling pathway. Figure 3 illustrates the DON-mediated signal transduction in the apoptotic process.

## 4. Occurrence and Prevention of DON

### 4.1. Occurrence of DON

DON, a naturally occurring metabolite, is synthesized by fungi belonging to *Fusarium* genus, notably *Fusarium graminearum*, *Fusarium asiaticum*, and *Fusarium culmorum* [101]. DON can occur at pre-harvest when the plants are growing, during harvesting, or during the post-harvest processing and storage. Recent studies found that mild temperatures and high humidity during flowering and maturation periods promote the growth of fungi [102,103]. Environmental conditions, such as temperature, moisture, water activity, substrate oxygen levels, pH, physical damage, competition, and fungal spores are essential in toxin growth and accumulation [104]. Previous studies have documented the specific conditions conducive to DON production. Peter Mshelia et al. [105] reported that *Fusarium verticillioides* and *Fusarium graminearum* isolated from maize cultivated on milled-maize agar could produce maximum DON content (0.22 mg/kg) in growth chambers for 21 days under specific environmental conditions, including 0.98 water activity, 30 °C, and 400–800 ppm of carbon dioxide (CO_2_). Belizán et al. [106] illustrated that *Fusarium graminearum sensu stricto* strains derived from wheat yielded the highest DON content (47.5 µg/g) in Petri dishes for 28 days under specific environmental conditions, including 0.995 water activity and 30 °C. Similarly, Rybecky et al. [107] utilized *Fusarium meridionale* isolated from soybeans in Argentina, pinpointing the optimal environmental conditions for DON occurrence at 0.965 water activity and 25 °C. Thus, DON production and accumulation is markedly influenced by weather conditions, emphasizing factors such as temperature and water activity. Apart from environmental conditions influencing *Fusarium* growth, some mycotoxin-producing saprophytic fungi, including *Aspergillus*, *Claviceps*, *Stachybotrys*, *Fusarium*, and *Penicillium*, can also affect *Fusarium* growth by thriving on plant residues and persisting on plant surfaces [108]. For example, *Fusarium verticillioides*, a member of the *Fusarium* fungi genus, was detected in maize stored for 2 years, within a temperature range of 10–40 °C and a relative humidity of 40–88% [109]. Hence, thorough cleaning of the harvested grain is crucial for minimizing DON levels in grains, as the highest concentrations are often found in broken kernels and fine particles [110].

### 4.2. Prevention of DON Production

Currently, there are two comprehensive strategies, encompassing a range of technologies and methods, utilized to mitigate DON contamination. The first strategy involves preventing DON production in crops by controlling DON-producing plant pathogens (*Fusarium* spp.), while the second strategy focuses on detoxifying or decontaminating cereals once they enter the food chain. Preventive strategies entailed pre-harvest measures, including the application of pesticides and the cultivation of disease-resistant plant strains, and the implementation of harvesting controls by adopting a suitable harvest schedule and minimizing mechanical injuries [111]. Post-harvest controls focused on ensuring crop storage in appropriate environmental conditions with a moisture content of less than 13% (a water activity of around 0.7) and at low temperatures (depends on different grain types), non-conducive to DON growth [112].

The meticulous selection of high-quality seeds is paramount in ensuring the robustness of ensuing crops, enabling them to withstand potential assaults from pests and diseases. Consequently, choosing fungal-resistant crops represented a viable strategy to mitigate mycotoxin contamination in cereals [113]. Moreover, with the advancing sophistication of genetic modification techniques, the manipulation of crops’ genetic code emerges as a potent tool to enhance resistance against mycotoxin contamination [114]. This genetic intervention may offer direct solutions by generating crops inherently resistant to fungal threats or indirect solutions, such as producing crops with elevated nutrient contents, thereby fortifying the resulting products against more aggressive treatments.

The choice of sowing date plays a pivotal role in determining the flowering date and the type of variety sown, concurrently impacting on *Fusarium* spore production and infection influenced by prevailing weather conditions. More frequent and severe DON instances were documented when the flowering date coincided with the release of *Fusarium* spores during the month of June in winter wheat [115]. Given that the timing of occurrences is a critical determinant of infection, alterations in the sowing date or the ripening process of the variety can substantially impact fungal infection and the contamination of mycotoxins [116]. In maize crops, a delayed sowing date (in the middle of May) elevated the risk of mycotoxin contamination compared to an earlier sowing time (at the beginning of April) [117]. Munkvold [118] similarly reported that an earlier planting date for maize in temperate regions frequently correlated with reduced risk. However, the variability in annual weather conditions could potentially undermine this advantage. In barley, an early sowing date at the beginning of May in Canada decreased the severity of DON contamination compared to a late sowing date at the middle or end of May [119]. In wheat, selecting an earlier sowing date, such as July 4 in Argentina, has been demonstrated to result in decreased mycotoxin contamination compared to a delayed sowing date, such as August 24, evidenced by reduced levels of DON, 3-ADON, and ZEA at 39.35 vs. 166.51 ng/g, 10.34 vs. 47.94 ng/g, and 249.46 vs. 1516.92 ng/g, respectively [120]. Therefore, it is advisable to schedule the sowing time of different plant species sensibly to mitigate mycotoxin contamination [121].

Cultivation management is pivotal in pre-harvest control, offering a means to mitigate the risk of *Fusarium* spp. contamination. The rotation of crops, explicitly alternating between wheat and legumes, has proven effective in controlling mycotoxin contamination in some instances [122]. Additionally, adhering to proper soil cultivation practices, such as adopting minimum or no-tillage technology, contributed to removing and isolating infected crop residues [123]. This, in turn, reduced the risk of mycotoxin contamination in subsequently cultivated crops. When inadequately regulated, improper water and nutrition supplies could create soil stress conditions conducive to the accumulation of mycotoxins [122]. Furthermore, implementing insect control measures has been suggested as an approach to diminish infection levels, as insects possess the potential to inflict damage on the external protection of grains and plant tissue. They might serve as a carrier of fungal spores between different agricultural areas [116].

Harvesting date exerts a pivotal role in determining the final concentration of certain mycotoxins in cereal grains. Notably, Kharbikar et al. [124] observed a 20% reduction in ZEA concentration in Solstice wheat when harvested three weeks earlier. Similarly, Xue et al. [109] observed that compared to an earlier harvest date, a delayed harvest date in New Liskeard between 14 September and 19 September in 1999 and 2000 and in Winchester on 5 October in 1999 and 2000 increased levels of mycotoxins in wheat, attributing this to a robust correlation between harvesting time and the incidence of *Fusarium* spp. However, it was worth noting that harvesting time did not significantly impact the DON concentration in wheat [124,125].

In an increasingly globalized and specialized market where grain products are seldom produced and consumed within the same community, it is crucial to store and transport grains under conditions that inhibit the growth of mycotoxin-producing fungi and other pathogens. To achieve this goal, it is recommended that grains should be stored at a low water activity below 0.90, maintained at a low temperature below 20 °C, and ideally stored at a low concentration of CO_2_ at around 400 ppm [126,127,128]. Interestingly, the detailed environmental conditions vary from grain to grain. Additionally, to prevent the potential spread of contaminants among distinct grain storage facilities, it is essential to refrain from mixing grains stored separately for extended periods. Despite these precautionary measures, the likelihood of some grains becoming contaminated remains. In cases of infection, farmers should discard the affected grains through burning or burial to prevent the spread of infection to healthy grains.

In recent years, the surge in data collected from diverse sources and advancements in physical computing and data analysis methods, particularly in machine learning, has enabled the development of increasingly sophisticated predictive models. These models have found applications in various agricultural domains, from weather prediction to predicting crop disease occurrences. Some software applications have been created to assist farmers in anticipating the risk of mycotoxin contamination [129,130]. However, owing to the dynamic interplay between mycotoxin occurrence, climate, and other biological factors, these modeling systems still exhibited relatively low accuracy [131]. The recent exponential growth in the acquisition of various data types holds the promise of training more refined models through machine learning, potentially offering an effective method for mycotoxin prediction in the near future.

## 5. Detoxification of DON

The substantial negative impacts of DON on swine health and growth performance have led to extensive studies on the detoxification of DON in feed and feedstuff. Typically, the feed industry employs physical, chemical, biological, and nutritional strategies as the primary approaches for detoxifying DON. However, each of the above strategies has its own strengths and weaknesses. Accordingly, this section will concentrate on methods for DON detoxification, delineating their respective merits and drawbacks.

### 5.1. Physical Strategies

The physical strategies employed for DON detoxification include sorting, washing, dehulling, heating, milling, extrusion, and irradiation. Table 2 presents an overview of frequently utilized approaches for the physical detoxification of DON.

Sorting involves separating grains or food into categories based on their shape, size, weight, image, and color. Sorting is a rudimentary processing method employed to remove *Fusarium* contamination due to its relative simplicity and the widespread availability of necessary infrastructure. Sorting could decrease 0.6–20 mg/kg DON in the sorted wheat to 51% of its original level using one-pass sorting [132]. However, for a lower concentration in unseparated wheat, 899.1–2442.4 µg/kg DON, sorting could only achieve a reduction of 30.85% [133]. According to these results, detoxifying DON in grains through sorting alone was relatively ineffective; however, employing multiple strategies, such as near-infrared spectroscopy and optical visual sorting, could enhance accuracy, achieving over 92% precision in identifying moldy corn and wheat kernels [132,149]. The mechanism underlying near-infrared spectroscopy and optical visual sorting involves discerning distinguishing characteristics between *Fusarium*-damaged and healthy kernels, primarily focusing on kernel morphology and color. *Fusarium* damage typically manifests as whitish or pinkish kernels with a dried-out appearance, serving as key indicators for detection. Specifically, Pascale et al. [150] observed that using an industrial optical sorting machine led to overall reductions in mycotoxins in raw coming maize ranging from 36% to 67% for 3.2–17.4 mg/kg DON, 67% to 87% for 0.66–4.46 mg/kg ZEA, and 27% to 67% for 2.52–6.54 mg/kg FUM, respectively. While it was confirmed that sorting grains by size, shape, density, or color might help remove mycotoxin contaminations from a grain batch, current methods lacked precision and often missed contaminated grains or removed non-contaminated grains [151]. Hence, sorting is applicable primarily to small-scale operations, inadequate for effectively eliminating mycotoxin-contaminated grain, and its relatively high error rate often leads to product losses.

Washing is another proper physical method applied in the grain industry that helps remove water-soluble mycotoxins from the surface of cereals. Trenholm et al. [152] noted that using a 1 mol/L sodium carbonate solution in the initial washing stage led to reductions of 72.3–74.3% for 16.1–23.9 mg/kg of DON and 80.9–87.3% for 0.89–1.58 mg/kg of ZEA in barley and corn. Similarly, Yener and Köksel [153] reported pressure washing wheat grains with water, followed by oven drying at 32 °C for 15 h, resulted in mycotoxin reductions, with a 30.3% for 11.6 µg/g of DON and 21.1% for 0.39 µg/g of ZEA in wheat; subsequent drying using infrared and microwave methods at a total power of 525 W and 1000 W for 30 min and 1 min further decreased concentrations by up to 89.0% and 82.5% for DON and ZEA, respectively. Additionally, employing 2 mg/kg chlorinated water, 0.5 M and 1.0 M sodium carbonate, and 0.75 M and 1.5 M sodium hydroxide solutions to the wheat samples for 1 min led to DON (11.6 µg/g) reductions ranging from 37.3% to 91.2% and ZEA (0.39 µg/g) reductions ranging from 31.6% to 83.6%. While washing has the potential to detoxify mycotoxins to some degree, the processes must be coupled with drying. Neglecting this precaution might lead to grain scaling, nutrient loss, or even heightened mycotoxin production due to water retention. This would require significant energy input as heat on a large scale, making it an expensive solution.

Dehulling is another essential processing step used in the agricultural industry, in which the outer hull of cereal grains is removed. It is often the case that *Fusarium* spp. colonization is localized in the bran fraction of grains. A prior study found that around 80% of the DON content in intact maize kernels resided within the pericarp and germ, with the pericarp containing 55%, and the germ containing 25% [154]. Thus, dehulling can also reduce DON contamination in cereals [155]. Trenholm et al. [156] found that employing a dehulling process led to reductions in the concentrations of DON and ZEA in ground barley, wheat, and corn from initial levels of 5.0–23.0 mg/kg and 0.5–1.21 mg/kg, respectively, to 34% in barley, 39% in dehulled and ground barley, 55% in wheat, and 69% in corn. Additionally, using an efficient Scott-strong dehuller reduced 5.0–23.0 mg/kg DON and 5.0–23.0 mg/kg ZEA in the grains by 40% to 100% and 13% to 19%, respectively. Pearling, a pre-milling procedure involving abrasion and friction, selectively eliminates bran layers from cereal grains, thereby preserving essential components such as the aleurone layer within the intact kernels [157]. Rios et al. [158] employed the pearling process to investigate the detoxification of DON in wheat, revealing a significant reduction in the initial DON level of 4.2 mg/kg, particularly following the removal of 10% of the grain tissue, resulting in a notable 45% decrease in DON levels. It is crucial to recognize that while complete dehulling could eliminate mycotoxins to some extent, it may also expose the nutrient-rich endosperm. Hence, if mycotoxins are not entirely removed from a given batch, it may result in further fungi growth due to the improved accessibility to nutrients.

Heating or thermal processing is another physical method that can be used to detoxify mycotoxins in cereals. One previous study reported that heating whole barley powder samples for 60 min at 160 °C, 180 °C, 200 °C, and 220 °C resulted in 8.75 µg/g DON decomposition to 48%, 79%, 96%, and 100%, respectively; and the detection of DON was conducted using gas chromatography-mass spectrometry (GC-MS), achieving a detection limit of 0.5 ng/mL [159]. DON reduction depends on temperature and processing time, with the most remarkable effects at the highest temperatures and longest processing time. Pronyk et al. [160] obtained comparable findings, indicating that a 6 min treatment with superheated steam at 185 °C resulted in a reduction of up to 52% in 15.8 mg/kg DON in wheat kernels, while no reduction in DON levels was observed with 2–15 min treatments at 110 °C and 135 °C. Based on these findings, it can be concluded that a minimum temperature above 160 °C is required for the degradation of DON. While high temperatures were an effective method of DON detoxification, there was concern that the process could degrade nutrients in the cereal grains [17]. Specifically, Liu et al. [161] showed that superheated steam at 265 °C efficiently reduced 3.3 mg/kg DON in the scabbed wheat samples by 77.5% and decreased 1.9 mg/kg DON in wheat flour by 60.5%, albeit inducing partial denaturation of protein and partial gelatinization of starch, subsequently influencing the rheological properties of dough and pasting characteristics of wheat flour. On an industrial scale, similarly to the washing process, this method demands considerable energy inputs, potentially resulting in heightened expenditures while concurrently impacting nutrient content in cereals.

Milling is one of the oldest feed/food processing techniques. Interestingly, although the milling process did not directly affect the mycotoxin levels in grains, it changed the distribution of mycotoxins among the different fractions rather than removing or eliminating DON levels or any other mycotoxins [162]. Substantial evidence indicates that milling can alter the distribution of mycotoxins in grains. Young et al. [163] employed milling techniques for the detoxification of DON in Ontario soft white winter wheat (0.58 mg/kg), revealing a fractionation process where DON levels increased in the outer kernel portions (bran, 0.98 mg/kg) and decreased in the inner flour portions (break flour, 0.28 mg/kg). Abbas et al. [164] also reported that using a milling process could modify the distribution of DON in DON-contaminated wheat (7.9–9.6 mg/kg), demonstrating that 21 mg/kg of DON was present in the bran, while only 1 mg/kg of DON was found in the break flour. Likewise, Zhang and Wang [165] discovered that wheat grains contained 4.68–36.72 µg/g of DON and 0.17–1.04 µg/g of D3G, while using a milling process resulted in a significant reduction of 79–90% in DON and 23–39% in D3G in milling flour, accompanied by an increase of 125–221% in DON and 259–440% in D3G in wheat bran, and an increase of 113–219% in DON and 263–337% in D3G in wheat shorts. It was noteworthy that milling decreased the overall concentration of DON but concurrently increased the level of D3G, possibly attributable to the binding of DON to starch during the milling process [165]. Thus, milling is not well suited to detoxification as DON and may result in secondary contamination. Moreover, reducing mycotoxins through milling is contingent on various factors, including the type of miller, milling speed, and moisture content of the raw materials [141,142].

Extrusion holds pivotal significance due to its widespread application in the food industry. Extrusion enhances product quality by modifying texture and increasing digestibility and influences the levels of mycotoxins in the final product [166]. Some studies have used extrusion techniques to investigate its detoxification effect on DON-contaminated grains. For instance, Wolf-Hall et al. [167] observed that the concentrations of DON in corn grits and dog food were 4.0 mg/kg and 3.86 mg/kg, respectively, and after extrusion at 100 °C for 5 min and then dried overnight at 60 °C could result in a reduction of 53% and 21% in the levels of DON. The difference in reduction rates observed in corn grits and dog food under identical temperature and duration conditions may be attributed to the distinct ingredient compositions of these two products. Moreover, the main factor for DON reduction appears to be temperature. Cetin and Bullerman [168] determined that extrusion processing at 200 °C resulted in a higher reduction of 23.5 µg/g DON in corn grits by 34% compared to 150 and 175 °C, while extrusion screw speeds of 70 and 140 rpm had no significant effects on DON reduction. Apart from temperature, some extrusion parameters factors influence the effectiveness of DON detoxification. Hajnal et al. [169] found that the optimal parameters for reducing the concentration of each investigated mycotoxin in naturally contaminated whole grain triticale flour were identified as follows: screw speed: 650 rpm; feed rate: 30 kg/h; moisture content: 20 g/100 g; these resulted in reductions of 9.5%, 27.8%, 28.4%, and 60.5% for 274.4 µg/kg DON, 2.86 µg/kg 3-ADON, 4.86 µg/kg 15-ADON, and 4.59 µg/kg HT-2, respectively. These above studies indicated that optimal reduction rates of mycotoxins in the final product were achieved by employing extrusion process parameters featuring medium screw speed, the highest temperature, the highest feed rate, and the lowest moisture content of raw grains. Notably, the extrusion of starchy foods could potentially impart adverse effects on the final product, including gelatinization, partial or complete destruction of the crystalline structure, molecular fragmentation of starch polymers, protein denaturation, and the formation of complexes between starch and lipids, as well as between protein and lipids [170]. The nutritional changes mentioned above in cereals might limit their application at industrial scales. Furthermore, in instances where raw grains are severely contaminated with mycotoxins, achieving maximum reduction in the content of each mycotoxin through the extrusion process is challenging.

Irradiation, called “cold pasteurization,” is a nonthermal method commonly applied in feed processing. In contrast to traditional pasteurization, which relies on heat, irradiation inactivates microorganisms at a lower temperature. Irradiation manifests in gamma (γ) and electron beams, with the effects produced by these two forms of radiation often identical. Higher γ-ray irradiation improved degradation efficiency [144]. The γ-ray irradiation at 10 kGy resulted in a 33% reduction in 10.0 mg/kg DON in soybeans [171]. Li et al. [172] observed a significant degradation effect of ^60^Cobalt γ-ray irradiation on pure 2 μg/mL DON in acetonitrile–water, achieving an 83% degradation efficiency when exposed to 20 kGy γ-ray irradiation. Meanwhile, complete degradation of 2 μg/mL DON in ultra-pure water occurred after 5 kGy γ-ray irradiation. The effectiveness of γ-irradiation in degrading mycotoxins depends on factors such as the type and concentrations of the mycotoxin in cereals, the matrix, moisture content, and the radiation dose and duration. Nevertheless, it was noteworthy that increased irradiation, while capable of degrading mycotoxins, could impact the nutrient levels of the grain [171]. Electron beam irradiation, generated by electron accelerators, has been studied for its capability to degrade DON. At 54.5 kGy of electron beam irradiation, 4.95 mg/kg DON in dry wheat decreased by 17.6%, whereas no reduction was observed in 2.0 mg/kg DON in dry distiller’s dried grains with solubles (DDGS) within the electron beam irradiation dose range of 2.6 to 51.8 kGy [173]. Consistent findings were reported by Kottapalli et al. [174], indicating that electron beam irradiation within the range of 0 to 10 kGy did not result in the degradation of 0.7–1.7 μg/g DON in raw barley. The efficacy of electron beam irradiation in degrading DON could be influenced by critical factors, including substrate type, irradiation dose, mycotoxin level and type, and water ratio [175]. Similarly to γ-ray irradiation, an increasing dose of electron beam irradiation detrimentally impacts the quality of cereals, leading to decreases in amylose content, essential and total amino acid contents, and starch crystallinity, as evidenced in corn samples treated with electron beam irradiation at 30 kGy [176]. Hence, experts from the FDA reported that foods irradiated at doses below 10 kGy were considered safe and healthy [177]. While the irradiation methods discussed above could decrease the DON content in contaminated cereals, factors such as expensive equipment and public concern regarding the chemical safety of ionizing radiation, as well as the toxicity of resulting derivatives and the potential for negative nutritional changes, continued to limit widespread application in feed.

Ultraviolet (UV) germicidal irradiation emerges as a promising method for mycotoxin elimination, as it has been successfully employed in post-harvest sterilization of agricultural products, including stored grain for foodstuffs or animal feed [145]. Exposing DON-contaminated moist corn silage to UV treatment (15 mW/cm^2^ at 254 nm UV-C wavelength) for 30 and 60 min resulted in a reduction of 60 μg/g DON by 22% and 21%, respectively, with the concentrations of α-tocopherol and β-carotene remaining relatively unaffected [178]. More recently, a substantial reduction of 97.3% and 75.4% in 2 mg/kg DON and 2 mg/kg ZEA on 0.1 mg of filter paper was achieved with a given UV-C dose of 15,000 mJ/cm^2^, and crucial parameters such as moisture content, protein content, and the percent of germination of maize kernels remained unaffected by UV-C treatment up to 5000 mJ/cm^2^ [179]. The effectiveness of UV treatment in reducing DON relies on the specific food matrix or substrate utilized. Doping materials modified with oxide semiconductors, such as TiO_2_, Fe_2_O_3_, etc., could effectively degrade DON under light conditions. For example, Wang et al. [180] demonstrated that dendritic-like α-Fe_2_O_3_ exhibited superior activity in the photocatalytic degradation of DON in an aqueous solution under visible-light irradiation (λ > 420 nm), achieving a reduction of 90.3% of DON with an initial concentration of 4.0 µg/mL within 2 h. Similarly, the TiO_2_ catalyst doped with 0.5% cerium demonstrated heightened photocatalytic efficacy in degrading DON in aqueous solution under ultraviolet light irradiation, resulting in the degradation of 96% of DON with an initial concentration of 5.0 mg/L within 4 h [181]. The degradation rate of DON through light treatment depends on the delivered light energy, which is influenced by some factors, including substrate matrix, the wavelength of light, exposure time, etc. Further experiments are essential to explore efficient UV irradiation methods for DON detoxification without compromising the nutritional quality and taste of feed, thereby enhancing the safety of grains for use in animal feed.

In addition to traditional methods for detoxifying DON, novel approaches, such as cold atmospheric plasma (CAP) and plasma-activated water (PAW), have garnered interest. CAP stands out as a novel physical method with numerous advantages over traditional methods, characterized by its cost-effectiveness, environmental friendliness, and minimal to no adverse effects on product quality [182]. This method relies on increasing a substance’s energy level, resulting in the transition from a solid state through the various states of matter, resulting in an ionized state of gas known as the plasma state [183]. The mechanisms of CAP decontamination are mainly ascribed to the highly reactive oxygen and nitrogen species (ROS and RNS) and ultraviolet radiation generated in plasma oxidation, resulting in a high degree of oxidation. CAP impacts the chemical structure of the mycotoxins, leading to their degradation. Applying high voltage CAP treatment at 85 kV for 20 min in a double barrier discharged system, when applied to suspensions containing 100 µg/mL of DON, yielded a reduction 99% in DON concentration [184]. Similar results were obtained by Hojnik et al. [185], who found that the application of CAP treatment at 10 kV in a surface barrier discharge system resulted in a 95.9% reduction in 27 µg/mL DON after an 8 min exposure. As CAP demonstrated highly effective detoxification efficiency in DON-contaminated aqueous solutions, numerous studies have explored its application in the analysis of DON-contaminated grains. Specifically, under optimal conditions, including a voltage of 100 V, frequency of 200 Hz, and duty cycle of 80%, double dielectric barrier charge CAP treatment for 25 min led to a 98.94% reduction in 0.5–5 µg/mL DON solution [186]. Feizollahi et al. [187] also reported that applying double dielectric barrier charge CAP treatment at 34 kV induced a 48% reduction of 37.0 µg/mL DON on barley grains in 6 min. Furthermore, CAP treatment in aqueous solutions efficiently reduced DON than in dry conditions [147]. While CAP technology exhibits superior potential to traditional methods, its current constraint lies in limited detoxification batch sizes. Achieving optimization and scaling to industrial levels necessitates a more comprehensive understanding of these processes.

Plasma-activated water (PAW), a byproduct of nonthermal plasma with water, emerges as a novel method for mycotoxin detoxification. Similar to CAP, the decontamination mechanisms of PAW are primarily attributed to highly biochemical ROS and RNS [188]. In the liquid phase, PAW facilitates the interaction between reactive chemistries and contaminant molecules, thereby holding the potential to comprehensively treat the surfaces of samples [189]. In recent years, numerous research groups have delved into the detoxification of DON using PAW. For instance, Qiu et al. [190] showcased a 58.78% degradation rate of 1.5–2.0 mg/kg DON in wheat samples through a 24 h treatment with PAW, which was produced at 50 kV for 10 min using a dielectric barrier discharge cold plasma generator, highlighting the significant roles of H_2_O_2_ and ozone as crucial contributors to DON degradation in PAW. Chen et al. [146] documented a 34.6–38.3% reduction in 1.45 mg/kg DON within 5–20 min of treating germinating barley with PAW. H_2_O_2_, ozone, nitrate, and low pH collectively serve as critical factors in the inactivation process within PAW. More recently, direct bubble spark discharge at 15 °C reduced 4.6–5.8 mg/kg DON in naturally infected barley grains by 57.3% within 30 min due to the actions of H_2_O_2_ and ozone [191]. Although PAW treatment has shown efficacy in detoxifying DON from grains, it was accompanied by certain limitations, including high costs, operational risks, and a lack of approved regulatory standards [192].

In summary, while current physical methods can reduce the content of DON in cereals to some extent, they are still regarded as the first step of all detoxification methods. Physical methods should be used in combination with other chemical or biological methods to achieve the required reduction in DON contamination in feed to impact pigs positively. Moreover, although some novel physical methods have been shown to detoxify mycotoxins effectively, the cost of equipment and changes in the nutritional composition of cereals will limit their application in industrial production.

### 5.2. Chemical Strategies

Compared to physical methods, chemical methods have inherent advantages, such as higher efficiency and lower equipment requirements. At present, the chemical detoxification of DON could be achieved using alkaline chemicals, organic acids, or ozone. However, the majority of these chemical methods have raised concerns regarding consumer health [193]. Hence, the EU prohibited using chemical treatments to reduce mycotoxins in feed and food materials in 2006. Table 3 presents an overview of frequently utilized approaches for the chemical detoxification of DON.

**Figure 4:**
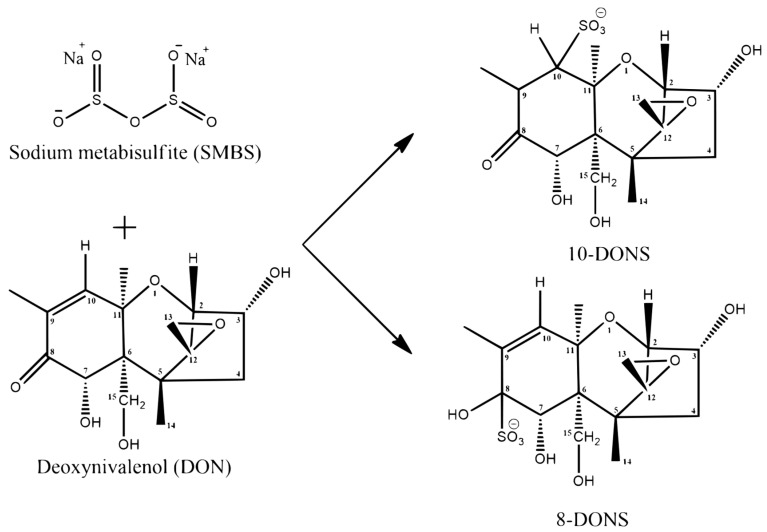
The reaction of deoxynivalenol (DON) with sodium metabisulfite (SMBS) and the molecular structures of the two DON sulfonate (DONS) products (8-DONS and 10-DONS) [212].

DON detoxification in contaminated cereals by chemical approaches was achieved using several mechanisms, including alkalization, reduction, hydrolysis, ammoniation, and oxidation [193]. Of these mechanisms, reduction is particularly promising as it can change DON’s molecular structure and biological activity. For instance, DON could be transformed into DON-sulfonate (DONS) by reducing agents such as sodium bisulfate (NaHSO_3_) and sodium metabisulfite (SMBS, Na_2_S_2_O_5_). SMBS can react with the C9-C10 double bond or C8 keto group of DON, resulting in the formation of two highly polar diastereoisomers of DONS derivatives, 8-DONS and 10-DONS [212]. The detailed reaction is shown in Figure 4. Previous studies have shown that DON concentration has a 50% inhibitory (IC_50_) effect on the proliferation of porcine PBMC, untransformed IPEC-1, and IPEC-J2, and human HepG2 cells, which were 1.18 μM, 1.33 μM, 2.97 μM, and 41 μM, respectively, while the IC_50_ values of DONS inhibiting the proliferation of these four cells were much higher than that of DON [213]. Thus, DONS seems to be significantly less toxic than DON, although these results from in vitro tests have yet to be replicated during in vivo experiments. Treatment with 10 g/kg of SMBS at a moisture content of 22% was applied to a wheat batch contaminated with 7.6 mg/kg of DON for 15 min at 100 °C, resulting in complete degradation of DON [197]. The study also demonstrated that the growth performance of piglets fed SMBS-treated moldy wheat was significantly superior to those provided untreated moldy wheat, although no significant difference was observed between the control group and the group fed moldy wheat treated with SMBS [197]. Moreover, when naturally contaminated maize was treated hydrothermally with the addition of 5 g/kg SMBS, 10 g/kg methylamine, and 20 g/kg calcium hydroxide for 30 min at 80 °C, an 91% reduction in 43.4 mg/kg DON was observed; and the detection of DON was conducted by liquid chromatography-mass spectrometry (LC-MS/MS), achieving a detection limit of 0.5 ng/mL [200]. While these results were promising, one concern was that alkaline conditions might hydrolyze unstable DONS derivatives back into DON [194]. A previous study conducted by Danicke et al. [197] did not detect DON in serum, which illustrated that hydrolyzation of DONS derivatives did not occur in the digestive system of piglets. Indicating that the transformation products were made stable in the stomach and were not hydrolyzed back to DON in neutral and weak alkaline conditions in the small intestine in pigs. Although in vitro chemical strategies such as SMBS have sound detoxification effects, one must pay attention to the newly generated DONS, including its potential to be converted back to DON under alkaline conditions and its adverse effects on the nutrition and flavor of feed.

Ozone, a gas characterized by its high oxidizing potential, has been evaluated for its capability to detoxify DON. Ozone is inherently unstable and can rapidly convert to oxygen without leaving any residue. In 1997, America recognized ozone as Generally Recognized As Safe (GRAS), and the FDA permitted its direct application to food [214]. The ozone treatment mechanism involved modifying the chemical structure of numerous mycotoxins, thereby reducing their biological activity as assessed in the employed bioassays [215,216]. Various research groups have explored the application of ozone for detoxifying DON in cereals. The concentration of 2.09–3.01 mg/kg DON in naturally contaminated wheat bran samples was reduced by 32% after ozone treatment at 62 mg/L for 240 min [217]. The content of 3.89 mg/kg DON in whole wheat flour samples decreased by 78.6% when treated with ozone at concentrations of 100 mg/L for 60 min [218]. In a recent study, Krstović et al. [219] observed that the application of an ozone level of 85 mg/L with 10% moisture content for 180 min resulted in the highest reductions in 11.26 mg/kg DON in ground corn samples by 42.8% than ozone levels of 40 mg/L and 99 mg/L. Similarly, in a study conducted by Sun et al. [216], applying a saturated aqueous ozone level of 80 mg/L to contaminated wheat, corn, and bran samples resulted in reductions of 2.18 mg/L, 2.93 mg/L, and 3.70 mg/L DON by 74.86%, 70.65%, and 76.21% within 10 min, respectively. The above evidence indicated that employing aqueous ozone and higher ozone levels was more effective in reducing DON than dry ozone and lower ozone levels. Nevertheless, implementing aqueous ozone and higher ozone levels on a large scale in industrial settings poses significant challenges. Furthermore, the determination of the safety of DON was hindered by the lack of knowledge regarding its ozone decomposition products, thereby restricting its feasibility for use in industrial applications [209]. Significantly, specific studies suggested that ozone treatment of contaminated cereals might result in a decline in the grains’ enzymes, amino acids, and other essential nutrients [220,221]. Hence, further research is essential to identify practical ozone methods that can effectively and safely detoxify DON while preserving the grain’s nutritional integrity.

Organic acids, such as citric acid and lactic acid, are frequently used in food and feed preservation, as well as feed processing, and have shown the capacity to improve the nutritional attributes of feeds. This was achieved by facilitating the degradation of anti-nutritive substances, such as phytate, and improving the utilization of phytate-bound phosphorus. A 92% reduction in 140 µg/kg AFLA content was observed in contaminated sorghum upon the addition of 8.0 mol/L citric acid at 4 °C for 72 h, and a 67% reduction in 140 µg/kg AFLA concentration was achieved with the addition of 8.0 mol/L lactic acid at 4 °C for 72 h [222]. One study also indicated a 97.2% reduction in 2.3 µg/kg AFLA content in contaminated rice with 1 mol/L of aqueous citric acid at 40 °C for 48 h [223]. Likewise, Humer et al. [224] found that applying 5% solutions of citric acid or lactic acid to treat contaminated feed samples with a ratio of 1.2:1 (*v*/*w*) for 48 h resulted in highest reduction in the 6.1 mg/kg DON by 45.9% and 37.7%, respectively. Notably, citric acid exhibits more efficient detoxification of DON and AFLA than lactic acid. Applying organic acid treatment is anticipated to detoxify DON in feed/food, but the underlying mechanism remains unclear.

Implementing chemical processing leads to the formation of new compounds, necessitating a thorough examination of byproducts to assess their toxicity. Chemical strategies for decontaminating DON have the potential to compromise the nutritional content of grains and impact their texture and taste, raising concerns for human health. Despite the favorable detoxification ability of SMBS, caution is warranted due to the potential conversion of its byproduct (DONS) back to DON under alkaline conditions, necessitating further safety evaluations in vitro. Concerns over consumer health have led to restrictions on the authorization of specific chemical methods, with the EU banning their application on cereal grains intended for human food in 2006. In the United States, ammonia is the sole approved chemical treatment method for AFLA detoxification. While these limitations prompt farmers to explore alternative solutions for managing DON contamination, some large-scale pig operations continue to rely on chemical approaches to DON decontamination, facilitating high-throughput production. The efficacy and safety of chemical methods in DON detoxification warrant comprehensive discussion to inform their judicious incorporation into industrial production processes.

### 5.3. Biological Strategies

Although the physical and chemical approaches mentioned above can remove or detoxify a significant portion of DON found within contaminated cereals, there are limitations, including ecological and economic problems, as well as concerns regarding how the treatments impact the nutritional value of food. As a result, many consider biological methods, such as using microbes or enzymes they produce, to be the most promising approach to degrade mycotoxins. Microbes and enzymes can metabolize DON into either less toxic or non-toxic products. In-depth investigations have been carried out on microbes and enzymes to delve into the mechanisms of detoxifying DON, which include various processes like de-epoxidation, oxidation, epimerization, glucosylation, glutathionylation, hydroxylation, and isomerization. This section provides a summary and discussion of microbes and enzymes capable of eliminating DON, and some fundamental detoxification mechanisms. Table 4 and Table 5 summarize the microbes and enzymes involved in DON detoxification.

One of the critical toxic sites in the structure of DON is the C12-C13 epoxide group. De-epoxidation is a reduction reaction that involves the opening of the 12-13-epoxy ring, transforming DON into its de-epoxide metabolite, DOM-1 [287]. The IC_50_ values of DOM-1 (83.0 mM) in 3T3 mouse fibroblasts demonstrated a 55-fold increase compared to DON (1.50 mM), as determined by the 5-bromo-2′-deoxyuridine (BrdU) assays [288]. Similarly, Pierron et al. [289] revealed that a 4 h exposure to 10 μM DON resulted in intestinal lesions in the explants, whereas treatment with DOM-1 under the same condition did not cause any impairment. The above results showed that DOM-1 was less toxic than DON in the intestinal explants. De-epoxidation of DON can occur in both anaerobic and aerobic conditions. Under anaerobic conditions, anaerobic microbes derived from the bovine rumen, fish gut, or chicken digesta and intestine were recognized for their capability of DON epimerization. Within the bovine rumen, the bacterial strains BBSH797 and *Coriobacteriaceum* DSM 11,798 could metabolize 25 mg/L DON into DOM-1 under anaerobic conditions at 37 °C for 48 h [225,226]. A microbial culture C133 from fish digesta could completely convert 50 µg/mL of DON into DOM-1 in full growth medium at 15 °C for 96 h incubation [227]. Likewise, microorganisms sourced from chicken digesta and intestine, including *Bacillus* sp. LS100 [228], *Clostridium* sp. WJ06 [229], *Eggerthella* sp. DII-9 [230], *Slackia* sp. D-G6 [231] and bacterial consortium YM-1 [232] exhibited the ability to metabolize DON into DOM-1 under various anaerobic environmental conditions. While these microbes demonstrated the capability to transform DON into DOM-1, their practical utility in production settings may be restricted, as it requires anaerobic conditions for efficient deoxygenation. Yet, under aerobic conditions, microbes from soil samples could transform DON into DOM-1. Islam et al. [233] documented that, under anaerobic conditions at temperatures ranging from 12 °C to 40 °C and pH levels of 6.0–7.5, six bacterial genera, namely *Serratia*, *Clostridium*, *Citrobacter*, *Enterococcus*, *Stenotrophomonas*, and *Streptomyces*, exhibited the ability to degrade 50 µg/mL of DON into DOM-1 for 60 h fully. Likewise, a bacterial consortium, PGC-3, derived from 14 soil samples, demonstrated the most robust and consistent activity in converting 100 µg/mL of DON into DOM-1 for 168 h under aerobic conditions [234]. Examination of the 16S rDNA sequences revealed that PGC-3 consisted of 10 bacterial genera, with *Desulfitobacterium* potentially playing a decisive role in de-epoxidation. Under aerobic conditions, PGC-3 displayed de-epoxidation activity across a broad range of pH values (5–10) and temperatures (20–37 °C). In a subsequent study by He et al. [235], it was intriguingly revealed that *Desulfitobacterium* sp. PGC-3-9 has been recently recognized for biotransforming 100 µg/mL of DON into DOM-1 for 168 h under anaerobic and aerobic conditions. It demonstrated substantial de-epoxidation capability for DON across a broad spectrum of pH values (6–10) and temperatures (15–50 °C). *Desulfitobacterium* sp. PGC-3-9 presents a promising option for detoxifying DON in cereals or serving as a feed additive in feed.

The C3-OH group stands out as another significant toxic site within the structure of DON. Documented alterations at C3 involved the oxidation and epimerization of the associated hydroxyl group, a process shown to diminish toxicity [16]. The outcome of DON oxidation at the C3-OH position is 3-keto-DON. The IC_50_ values of 3-keto-DON (4.22 and 3.67 µM) in Caco-2 cells and 3T3 mouse fibroblasts were determined to be 3.05 and 4.57 times higher than those of DON (1.38 and 0.804 µM), respectively, using the 3-(4,5-dimethylthiazol-2-yl)-2,5-diphenyltetrazolium bromide (MTT) and BrdU assays [16]. Shima et al. [290] obtained similar findings, concluding that the immunosuppressive effect of 3-keto-DON was 90% less pronounced than that of DON, as evidenced by alterations in mitogen-induced and mitogen-free mouse spleen lymphocyte proliferation. Many studies have identified that some bacteria with the capacity to convert DON into 3-keto-DON. A *mixed culture D107* was isolated from soil in farmland, demonstrating the ability to oxidate 200 µg/mL of DON for 5 days at 20 °C in aerobic conditions, with the principal product being the formation of 3-keto-DON [236]. Wang et al. [237] successfully isolated *Devosia insulae* A16, a short, rod-shaped, Gram-negative bacterial strain from the soil, demonstrating its capacity to oxidize DON, 3-ADON, and 15-ADON into 3-keto-DON and further establishing its efficacy in eliminating 84.7% of DON at a concentration of 20 mg/L under aerobic conditions at 35 °C and pH 7.0–8.0 for 24 h. Wang et al. [239] conducted screening of the bacterial consortium C20 isolated from wheat, illustrating its capability to enzymatically degrade DON, 3-ADON, and 15-ADON into 3-keto-DON, and successfully established its efficiency in degrading 70 μg/mL of DON at 30 °C and pH 8 for a 5-day incubation period. C20 consisted of various bacterial genera, notably *Methylophilus*, *Ancylobacter*, and *Devosia*, which exhibited significant increases in abundance in cultures with elevated concentrations of DON. Moreover, a synthetic bacterial consortium, including *Devosia* sp. A8 and *Paracoccus yeei* A9 exhibited effective degradation of 10 μg/mL of DON, 10 μg/mL of 3-ADON, and 10 μg/mL of 15-ADON at 30 °C and pH 8.0–9.0 for 72 h, achieving degradation rates of 100%, 49.76%, and 97.39%, respectively, with 3-keto-DON identified as the primary product [242]. Interestingly, in the synthetic bacterial consortium, *Devosia* sp. A8 assumed a primary role in the degradation of DON and its acetylated derivatives; however, *Paracoccus yeei* A9 contributed electrons to facilitate DON degradation by *Devosia* sp. A8. Similarly, Wang et al. [243] characterized a dual-member bacterial consortium comprising *Pseudomonas* sp. SD17-1 and *Devosia* sp. SD17-2, which exhibited efficient conversion of 50 μg/mL of DON into 3-keto-DON at 30 °C for 72 h and a pH range of 8.0–9.0. These two bacteria also worked in collaboration to accomplish the oxidation of DON. The above outcomes offer a novel concept that the bacterial consortium enables individual bacteria to fulfill their roles, resulting in an enhanced detoxification effect of DON or other mycotoxins.

The product of DON epimerization at the C3-OH position is 3-epi-DON. The IC_50_ values of 3-epi-DON (493 and 949 µM) in Caco-2 cells and 3T3 mouse fibroblasts were determined to be 357 and 1181 times higher than those of DON (1.38 and 0.804 µM), respectively, using the MTT and BrdU assays [16]. Zhang et al. [251] demonstrated that *Nocardioides* sp. ZHH-013, a Gram-positive, aerobic, rod-shaped bacterium isolated from soil samples with the capacity to degrade DON, by converting 168.74 μM of DON into 3-epi-DON over a 14-day incubation period at 30 °C, with 3-keto-DON serving as a crucial intermediary in the C3-OH epimerization pathway. Similar findings were achieved by He et al. [247], where they observed that a Gram-negative, aerobic, oval to rod-shaped bacterium named *Devosia mutans* 17-2-E-8 could degrade 100 μg/mL of DON into 3-keto-DON and 3-epi-DON under aerobic conditions at a spectrum of temperatures 25–30 °C and a pH range of 6.0–8.0, with 3-epi-DON being the primary product. Nevertheless, Ikunaga et al. [246] were the first to document that the *Nocardioides* WSN05-2, a Gram-positive, aerobic, non-spore-forming, coccal-shaped bacterium isolated from soil samples, could convert 1000 μg/mL of DON from culture medium into 3-epi-DON and an unidentified compound after incubation for 10 days. Then, they identified the previously unknown 3-epi-DON as an intermediate in the degradation process of DON. Wang et al. [248] also noted that *Paradevosia shaoguanensis* Strain DDB001, an obligately aerobic, Gram-negative, non-spore-forming and motile bacterium isolated from soil samples, could completely convert 200 mg/L DON into 3-epi-DON in complete growth medium at 30 °C, pH 7 and 0–2% NaCl for 48 h, with the intermediate 3-keto-DON going undetected. Lately, research groups have extensively explored bacterial consortia. For example, mixed cultures obtained from soil samples, including *Acinetobacter*, *Leadbetterella*, and *Gemmata*, could convert 100 μg/mL of DON into the major byproduct 3-epi-DON at 28 °C for 7 days [249]. Subsequently, the authors added naturally DON-contaminated wheat (7.1 μg/mL DON) to the mixed cultures, observing the near-complete degradation of DON into 3-epi-DON. Likewise, Gao et al. [252] reported that the combined action of *Pseudomonas* sp. B6-24 and *Devosia* strain A6-243 in a mixed culture facilitated the completely biotransformation of 100 μg/mL of DON and 3-keto-DON into 3-epi-DON through epimerization at temperatures of 16–37 °C and pH 7.0–10 for 48 h, with 15–30 μM pyrroloquinoline quinone (PQQ). *Pseudomonas* sp. B6-24 contributed to generating PQQ, while *Devosia* strain A6-243 played an essential role in the epimerization of DON.

Enzymatic degradation of DON is more targeted and efficient than microbial processes. Successful cloning and expression of genes encoding oxidases for DON and reductases for 3-keto-DON have been accomplished. The bacteria strain *Devosia mutans* 17-2-E-8, isolated from soil samples, demonstrated the ability to convert DON into 3-keto-DON and 3-epi-DON [247]. In subsequent investigations by Carere et al. [260,265], it was revealed that the detoxification mechanism employed by *Devosia mutans* 17-2-E-8 for DON involved a two-step epimerization process. Each stage of this process necessitated the involvement of a specific enzyme: DepA, recognized as a dehydrogenase, was tasked with converting DON into 3-keto-DON, while DepB, identified as an NADPH-dependent dehydrogenase, played a crucial role in the reduction of 3-keto-DON to 3-epi-DON. Likewise, He et al. [261] identified an aerobic *Devosia* strain, D6-9, in soil samples, which exhibited the capability to degrade 500 μg/mL DON into 3-keto-DON and 3-epi-DON at 45 °C and pH 6 for 2 h. Following genome and transcriptomes analysis, it was revealed that three essential genes played a role in the epimerization of DON: one gene encoded a quinone-dependent DON dehydrogenase (QDDH), which catalyzed the oxidation of DON to 3-keto-DON, while the other two genes encoded NADPH-dependent aldo/keto reductases (AKR13B2 and AKR6D1), facilitating the conversion of 3-keto-DON into 3-epi-DON. Notably, both DepA and QDDH were classified as PQQ-dependent dehydrogenases involved in the oxidation of DON. Li et al. [263] identified a sorbose dehydrogenase (SDH) with DON oxidation activity, dependent on PQQ, and demonstrated its applicability to degrade 15 μg/mL DON into 3-keto-DON across various conditions, including temperatures from 10–45 °C and pH levels of 4–9. In a recent study, Shi et al. [264] identified YoDDH, a DON-degrading enzyme derived from *Youhaiella tibetensis*, which displayed DON oxidation activity reliant on PQQ, demonstrating its largest activity at pH 4.5 and 40 °C, and exhibiting the capability to degrade 100 μM of DON into 3-keto-DON by 73% under above optimal conditions. However, Qin et al. [262] observed an additional deoxynivalenol dehydrogenase (DDH) capable of oxidizing 200 µg/mL of DON into 3-keto-DON by 90.5% across a broad pH range (6.0–11.0) and temperatures (20–45 °C), demonstrating DON oxidation activity independent of PQQ. In the process of DON epimerization, aside from DepB, AKR13B2, and AKR6D, Dep_BRleg_ (AKR18) exhibited the ability to convert 3-keto-DON into 3-epi-DON [266]. All these enzymes served as NADPH-dependent reductases, catalyzing the conversion of 3-keto-DON into 3-epi-DON.

Apart from bacteria and enzymes, antagonistic microorganisms also play a crucial role in inhibiting the development and DON production of toxigenic pathogens. *Trichoderma* strain exhibited a self-protective mechanism akin to that of plants, enabling them to detoxify 57 μg/g DON into D3G by 70% to 88% after incubation at 25 °C for 14 days when in competition with *Fusarium graminearum* [253]. Similarly, in the presence of *Fusarium graminearum*, the mycoparasite *Clonostachys rosea* ACM941 demonstrated the capability to bio-convert 125–500 μg/mL of 15-ADON into 15-AD3G through glycosylation at 25–28 °C for 10 days [254]. D3G is a masked mycotoxin formed through the enzymatic binding of glucose to DON within the plant [291]. The glycosylation of DON is recognized as a pivotal detoxification mechanism in plants, playing a crucial role in preventing *Fusarium*-related diseases. In the investigation of the toxicity of DON and D3G, Pierron et al. [67] illustrated that the IC_50_ value of D3G in Caco-2 human cells was 10 µM, which was notably 7.7 times greater than that of DON, estimated at 1.30 µM under identical experimental conditions. Enzymes belonging to the UDP-glycosyltransferases (UGTs) family were the primary agents through which host plants converted 10 mg/L DON into D3G in spikelets [281]. Currently, UGTs have predominantly been sourced from plants and are extensively discussed in the literature. UGTs responsible for DON glycosylation in various plant species included UGT12887 [273], TaUGT4 [275], Traes_2BS_14CA35D5D [279], TaUGT-2B and TaUGT-3B [280], and TaUGT6 [282] from *Tricum aestivum*, HvUGT13248 [274], HvUGT13248 [278], UGT13248 [284] from *Hordeum vulgare*, UGT73C5 [272] and TaUGT5 [281] from *Arabidopsis thaliana,* Bradi5g03300 [276] from *Brachypodium distachyon*, Os79 (Os04g0206600) [277] from *Oryza sativa*, and CrUGT3, CrUGT6 and CrUGT9 from *Clonostachys rosea* [285]. Leveraging the UGTs family presents a promising strategy for imparting resistance against *Fusarium* head blight.

In summary, compared with physical and chemical methods, the biological methods of DON detoxification show good prospects for application. However, biological methods have their limitations. For example, the growth of microorganisms, fungi, and their associated enzymes is relatively slow, so when applied as a treatment for DON detoxification, the contaminant concentration ought not to be too high; thus, the methods should be used in combination with chemical and physical methods as pre-treatment. In industrial production, this method can be used as a supplement to physical and chemical methods to minimize the negative impact of DON on pigs.

### 5.4. Nutritional Strategies

Despite the advancement of diverse in vitro detoxification techniques, including physical, chemical, and biological approaches, aimed at reducing DON concentrations in feed and agricultural products, the inherent physical and chemical stability of DON remains a significant barrier to complete degradation, thereby posing a persistent risk to animal health. Consequently, a range of in vivo nutritional strategies have been explored to further mitigate the impacts of DON on pig health. This section presents the detoxification effects of various nutritional strategies in pigs at different DON levels, providing tailored solutions for improving industrial pig production.

#### 5.4.1. DON Levels Below 1.0 Mg/Kg in Diets

The FDA and AAFC have established an upper limit for acceptable levels of DON in complete feed for swine, with a recommended threshold of 1.0 mg/kg [26,27]. Thus, diets containing less than 1 mg/kg DON are deemed inadequate for maintaining pig health [292]. However, a prior study reported that piglets fed diets contaminated with 0.9 mg/kg DON experienced weight loss and adverse effects on duodenal and jejunal morphology [45]. Several studies have sought to mitigate the effects of dietary DON levels below 1 mg/kg in pigs through some in vivo nutritional strategies. As discussed above, biodegradation is an effective, targeted, and environmentally friendly approach for mitigating the harmful effects of mycotoxins in feed. A previous study found that adding 1.5 g/kg of a mycotoxin-degrading enzyme, which includes esterase, epoxidase, and peptidase activities, to 1 mg/kg DON-contaminated diets for weaning piglets for 42 days could partially or completely mitigate the toxic effects of mycotoxins including growth performance, serum biochemical parameters, alveolar macrophage activity, antibody titters, cytokine secretion profiles, and histopathological observations [293]. Moreover, in a study by Shi et al. [294], two bacterial strains, *Bacillus subtilis* ANSB01G and *Devosia* sp. ANSB714, were administered at a concentration of 1 × 10^9^ CFU/g to diets contaminated with 0.796 mg/kg DON for 28 days, which resulted in significant increases in ADG, reductions in vulva sizes, and decreased some inflammatory cytokine levels in the plasma of gilts. SMBS-based feed additives have been successfully used in diets with high DON levels, and Shawk et al. [295] reported that a product called Defusion, comprising 92% SMBS along with organic acids, fermentation products, and supplemental vitamins and amino acids, could be effectively incorporated into DON-contaminated diets. The study found that pigs fed 2.5 g/kg or 5 g/kg Defusion or 2.5 g/kg or 5 g/kg SMBS during different growth phases exhibited improved growth performance when consuming diets containing less than 0.5 mg/kg of DON. Rapamycin (RAPA) and chloroquine (CQ) are two reagents that can modulate autophagy in animals. In a study by Liao et al. [296], the addition of 1 mg/kg RAPA or 10 mg/kg CQ to diets contaminated with 1.0 mg/kg DON demonstrated that pigs fed CQ alongside DON-contaminated diets exhibited improved growth performance and enhanced intestinal barrier integrity. Conversely, pigs fed RAPA alongside DON-contaminated diets showed increased intestinal autophagy, exacerbated inflammatory responses, and damage to the intestinal mucosa and permeability, which ultimately led to reduced growth performance. Taken together, it can be observed that different nutritional strategies at dietary DON levels below 1 mg/kg exert varying detoxification effects.

#### 5.4.2. DON Levels Between 1 Mg/Kg and 3 Mg/Kg in Diets

Low dietary concentrations (1.0 to 3.0 mg/kg) of DON in pigs were associated with decreased weight gain, anorexia, and immune changes [34,41,297,298,299]. To mitigate the health issues in pigs caused by DON, numerous studies have explored various in vivo nutritional detoxification strategies. These strategies include the use of binders or adsorbents, microbial or enzymatic biodegradation, plant extracts, DON-reactive chemicals, amino acids, and other approaches.

Microbial and enzymatic biodegradation represents an effective, targeted, and environmentally sustainable strategy for mitigating the detrimental effects of DON in pig feed. Currently, various microorganisms and enzymes exhibit differing levels of detoxification efficiency for DON in pig feed, particularly when its concentration ranges from 1 to 3 mg/kg. Li et al. [229] reported that daily supplementation of 30 mL of *Clostridium* sp. WJ06 with unknown concentration to diets contaminated with 1.90 mg/kg DON for 35 days improved growth performance, reduced the relative organ weights of the liver and kidney, and enhanced the integrity of the intestinal barrier in growing pigs affected by DON-contaminated diets. In a similar manner, Li et al. [298] demonstrated that supplementing drinking water with 1 × 10^8^ CFU/g of *Devosia* sp. ANSB714 for 18 days in growing pigs when fed diets contaminated with 2.85 mg/kg DON enhanced growth performance, improved immunological parameters, and bolstered antioxidant functions. However, several experiments have produced differing results. Dänicke and Döll [300] found that supplementing 2.75 mg/kg DON-contaminated diets in piglets with a probiotic additive containing *Bacillus subtilis* and *B. licheniformis* (1:1) at a concentration of 2.3 × 10^6^ CFU/g for 35 days did not mitigate the performance-suppressing effects of DON. In recent years, combinations of plant extracts and microorganisms have been developed as nutritional strategies in animal husbandry to address these issues by effectively mitigating the toxic effects of mycotoxins while enhancing nutrient availability and improving animal health. Specifically, glycyrrhizin, an extract from glycyrrhiza, has been shown to alleviate DON-induced oxidative stress, inflammation, and apoptosis in IPEC-J2 cells [301]. Subsequently, several studies have demonstrated that supplementing diets contaminated with 1.04 mg/kg DON in piglets for 28 days with a combination of 400 mg/kg glycyrrhizic acid, 1 × 10^6^ CFU/g *Enterococcus faecalis*, and 1 × 10^6^ CFU/g *Saccharomyces cerevisiae* improved growth performance, enhanced intestinal health, and mitigated DON-related liver damage and oxidative stress [302,303,304]. The underlying mechanism likely involves compound microorganisms in the combination degrading DON into less toxic substances while modulating the gut microbiota, with glycyrrhizic acid providing protection to organs and tissues from DON-induced damage. Despite the combination product’s potential to detoxify DON in vivo, the dietary levels of DON in these studies were restricted to 1.04 mg/kg, which was close to the regulatory threshold in pigs; thus, further experiments are required to evaluate the efficacy of this product for pigs subjected to higher concentrations of DON. The varying detoxification effects may be attributed to the inability to consistently preserve the activity of microorganisms or enzymes in animal feeds, highlighting the need to safeguard their activity and to further investigate the optimal inclusion levels. Moreover, the safety of these microorganisms or enzymes in animals should be thoroughly evaluated before they are introduced into industrial use [305].

Mycotoxin binders are large-molecular-weight compounds that bind to mycotoxins in animal feed, forming stable complexes that resist dissociation throughout the gastrointestinal tract, thereby preventing mycotoxin absorption in target organs and allowing for their excretion in feces, effectively mitigating the adverse impacts of mycotoxins [306]. Mycotoxin binders can be categorized into three sub-groups: inorganic compounds, organic compounds, and synthetic compounds [307]. Inorganic absorbent binders investigated for detoxifying DON in pigs include aluminosilicates, bentonites (montmorillonites), and hydrated sodium calcium aluminosilicate (HSCAS). Aluminosilicates, the most abundant group of rock-forming minerals, are characterized by a basic structural unit composed of interconnected silica tetrahedral and aluminum octahedral sheets, both of which are bonded to oxygen and hydroxyl groups [306]. Aluminosilicates are unable to ameliorate the adverse effects of DON in animal feed. In a recent study, Mwaniki et al. [38] illustrated that the addition of 1 g/kg purified hydrated sodium calcium aluminosilicate, Polaris (Probiotech International, Saint-Hyacinthe, Quebec, Canada), and 1 g/kg hydrated sodium calcium aluminosilicate, Epsilon-5 (Agri-Nutrient Solutions, Innerkip, ON, Canada) to a 2.86 mg/kg DON-contaminated diet in pigs for 28 days was ineffective in improving growth performance. Bentonites, often referred to as smectites due to their predominant mineral composition, are phyllosilicate clays characterized by a layered crystalline microstructure, and their adsorption effectiveness is influenced by both the montmorillonite content and the interchangeable cations present [308]. Recently, Shin et al. [309] demonstrated that the addition of 2.5 g/kg bentonites to 1.6 mg/kg DON-contaminated in pigs for 9 days could decrease the apparent ileal digestibility of zinc, arginine, isoleucine, threonine, and asparagine. Diet designers should carefully select mycotoxin binders or adsorbents to mitigate the effects of DON on pigs. HSCAS are frequently used in animal feed as anti-caking agents and function as enterosorbents by selectively binding to AFLA in the gastrointestinal tract, thereby reducing their bioavailability and associated toxicity [310]. Interestingly, Liu et al. [299] and Zhang et al. [311] found that the supplementation of 0.5 g/kg of a modified HSCSA binder Amdetox^TM^ (Jiangsu Aomai Biotechnology, Nanjing, China) in diets contaminated with 3.0 mg/kg DON for 28 days effectively mitigated DON-induced effects on the growth performance of piglets, improved intestinal flora balance, and enhanced thymic histopathology, apoptosis, redox status, and inflammatory responses. The observed results may be attributed to the modified HSCAS product, which is based on natural bentonite modified with cetylpyridinium chloride and intercalated with yeast beta-glucan; this modification significantly increases particle spacing by replacing interlayer cations and water molecules with the modifier, transforming the particle surface from hydrophilic to hydrophobic, thereby enhancing the adsorption capacity and lipophilic hydrophobicity of the modified HSCAS, ultimately contributing to improved immunity in animals [311]. While certain inorganic adsorbents could partially alleviate the effects of DON in pigs, it is important to recognize that clays may also absorb essential micronutrients, thereby reducing the bioavailability of minerals and potentially impairing nutritional balance [308]. Organic adsorbent binders explored for detoxifying DON in pigs include yeast cell walls, micronized fibers, and various bio-sorbents. Yeast cell walls, primarily composed of proteins, lipids, and polysaccharides—predominantly glucans and mannans—offer a diverse range of adsorption sites for mycotoxins, utilizing various binding mechanisms, including hydrogen bonding, ionic interactions, and hydrophobic forces, through interactions between mycotoxins and the functional groups on the cell wall surface [312]. Yeast cell walls have demonstrated significantly greater adsorption capacities across a broader spectrum of mycotoxins including ZEA, OTA, and FUM, not DON [313,314,315]. In a previous study, Shehata et al. [316] found that the addition of 5 g/kg modified yeast cell walls, or 5 g/kg of a combination of modified yeast cell walls and bentonite, to 1.73 mg/kg DON-contaminated diets in growing pigs for 28 days did not significantly contribute to the detoxification of DON. Similarly, Holanda and Kim [317] illustrated that supplementation of 2 g/kg yeast cell wall (MegaFix, ICC Brazil company, São Paulo, Brazil) to 1.9 mg/kg DON-contaminated diets in pigs for 35 days did not enhance growth performance, but it improved hepatic health, elevated glucose levels, and mitigated DON-induced gut damage. Then, a postbiotic yeast cell wall product developed by Alltech Inc., which consists of hydrolyzed yeast cell walls from *Saccharomyces cerevisiae*, organic acids (n-butyric acid), vitamins (ascorbic acid), and essential oils (rosemary extract), has also demonstrated partial improvement in the health of piglets exposed to diets contaminated with 2 mg/kg DON, although it did not affect growth performance [318]. Moreover, in the swine industry, an investigation into two commercial yeast cell wall products, Biofix (Biomin Inc., DSM-firmenich, Heerlen, The Netherlands) and Cel-can (VAST Inc. Mason city, IA, USA), in diets contaminated with 1.5–3 mg/kg DON in pigs revealed that neither product was effective in detoxifying DON [319]. Micronized fibers, derived from various plant materials such as cereals and legumes, are primarily composed of cellulose, hemicellulose, and lignin, and have been utilized as mycotoxin adsorbents due to their favorable gut adsorption properties and enhanced fecal excretion [320]. No studies have examined the adsorption capacity of micronized fibers for DON in pigs. In relation to bio-sorbents, apple pomace, which contains high levels of fibers and pectin, has been investigated as a mycotoxin adsorbent in pigs by incorporating it into diets contaminated with 2.1 and 3.1 mg/kg DON for 35 days, with authors suggesting that the addition of 80 g/kg pomace mitigated the adverse effects of DON [321]. Similarly to inorganic adsorbents, while some organic adsorbents could partially mitigate the adverse effects of DON on swine, it is important to acknowledge that these absorbents may also absorb essential micronutrients, thereby reducing the bioavailability of minerals and potentially compromise nutritional balance. Furthermore, excessive supplementation may pose limitations to their practical application in the industry. Synthetic adsorbents, such as modified clays, have been investigated for their potential to detoxify DON in pigs. Modified clays can overcome their limitations by altering their surface properties through the exchange of structural charge-balance cations with high-molecular-weight quaternary amines, leading to increased hydrophobicity [322]. However, a previous study reported that the addition of 1.7 and 5 g/kg algae-modified montmorillonite clay to 1.5 and 3 mg/kg DON-contaminated diets in pigs for 21 days did not mitigate the harmful effects of DON [323]. Taken together, while mycotoxin binders may effectively detoxify certain mycotoxins, they do not appear to be effective against DON; therefore, further research is needed to assess their potential use and impact on nutrient availability in diets.

As discussed above, the addition of microorganisms, enzymes, or mycotoxin binders in animal feeds does not consistently prove effective against chemically diverse mycotoxins, such as DON. Some research has utilized nutritional interventions to ameliorate intestinal abnormalities and growth retardation caused by the ingestion of mycotoxins. A diverse range of nutrients, including amino acids, trace element amino acid complexes, plant extracts, and organic matter, has been thoroughly investigated for their capacity to mitigate organ toxicity induced by DON [324]. Indeed, arginine-family amino acids, particularly arginine and glutamine, play crucial regulatory roles in swine nutrition by influencing nutrient metabolism and immune response, with studies showing that dietary supplementation with these amino acids can enhance porcine intestinal immunity and growth performance [325,326,327]. Yin et al. [328] illustrated that supplementing 10 g/kg L-arginine to 3 mg/kg DON-contaminated diets in growing pigs for 60 days effectively alleviated antioxidant system imbalances, mitigated intestinal abnormalities, and attenuated whole-body growth depression. In a similar vein, Duan et al. [329] demonstrated that the addition of 20 g/kg glutamate to 3 mg/kg DON-contaminated diets in growing pigs for 60 days yielded positive effects on growth performance and ameliorated the antioxidant system imbalance and intestinal abnormalities induced by DON. Taurine, a β-amino acid characterized by its natural sulfur-containing structure, is one of the most abundant free amino acids in mammalian tissues, including humans and most animals, and although it is not involved in protein synthesis, it remains an essential amino acid for growth and development due to its diverse physiological functions, which include antioxidant, anti-inflammatory, and osmoregulatory properties [330]. The addition of 3 and 6 g/kg taurine to 3 mg/kg DON-contaminated diets in piglets for 23 days demonstrated that, although 3 g/kg taurine did not enhance growth performance, it effectively alleviated DON-induced liver injury, as indicated by reduced pathological changes and improved serum biochemical markers [331]. Selenium (Se), an essential trace element, exhibits potent antioxidant, anticarcinogenic, and detoxification properties, and serves as a critical component of selenoproteins such as glutathione peroxidase, thioredoxin reductase, and iodothyronine deiodinase [332]. Organic Se refers to a group of Se chelates in which ligands donate electron pairs to Se, with amino acids commonly serving as dietary ligands in the synthesis of these Se chelates. Amino acid-chelated Se can be absorbed through peptide or amino acid transport systems, leading to enhanced digestibility and bioavailability of Se in animals. Selenomethionine (Se-Met), an organic selenium compound, is noted for its superior biological functions, mediated through selenoproteins, due to its structural similarity to methionine, where selenium replaces sulfur in the methionine molecule [333]. In a recent study, Zhong et al. [334] depicted supplementing 5 g/kg Se-Met to 3 mg/kg DON-contaminated diet in piglets for 28 days alleviated liver dysfunction, oxidative injury, and apoptosis through enhancing antioxidant capacity and inhibiting the JNK MAPK pathway. Plant extracts, composed of secondary metabolites responsible for a plant’s odor and color, have garnered significant interest for their potential biological functions, including antiviral, antimicrobial, antioxidant, and anti-inflammatory effects [335,336]. Several plant extracts have been studied for their potential to detoxify DON in pigs [337,338,339]. Resveratrol, one of the extensively studied plant extracts, has been demonstrated to effectively alleviate oxidative stress induced by DON in weaned piglets fed 2.65 mg/kg DON diets, while also reducing mitochondrial damage and improving intestinal morphology, ultimately enhancing growth performance of piglets [337]. Similar to resveratrol, hesperidin is a naturally occurring flavanone glucoside primarily extracted from various citrus fruits, and it possesses several beneficial effects, including antioxidants, anti-inflammatory, anti-tumor, neuroprotective, and immunoregulatory properties [338]. In a recent study, Li et al. [339] outlined that adding 0.3 g/kg hesperidin to 1.5 mg/kg DON-contaminated diets in piglets for 21 days could attenuate oxidative stress and intestinal injury. In addition to amino acids, trace element amino acid complexes, and plant extracts, organic matter such as humic substances have been proposed to prevent the absorption of toxic metabolites from the gut lumen in animals when administered orally [340]. However, the addition of 5 g/kg humic substances to 3.0 mg/kg DON-contaminated diets in pigs for 35 days did not prevent the absorption of DON [341]. In brief, some nutritional interventions may effectively mitigate the adverse effects of DON in pigs, necessitating further trials to determine their mechanism in reducing DON toxicity and identifying the optimal dosage, while also considering the cost as a significant factor. Additionally, if the DON level in the feed is higher than 3 mg/kg, the optimal levels of these nutrients need to be verified by more animal trials.

Compounds such as sulfite-reducing agents can directly detoxify DON in animals through reactive interactions with mycotoxin. Sulfite-reducing agents, including sodium sulfite (Na_2_SO_3_), sodium bisulfite (NaHSO_3_), and SMBS, showed a good application prospect and could react with DON at C9-C10 double bond or C8 keto group [212]. Several studies have investigated the detoxification ability of SMBS in pigs exposed to DON-contaminated diets. Frobose et al. [319] illustrated that the addition of 2.5 and 5 g/kg SMBS to diets contaminated with 3 mg/kg DON in pigs for 27 days enhanced the growth performance. In a similar manner, Mwaniki et al. [38] found that adding 3 g/kg SMBS to 2.86 mg/kg DON-contaminated diets in nursery pigs for 28 days effectively reduced DON levels to 1.21 mg/kg during feed pelleting and improved the growth performance, alleviated intestinal oxidative stress, and enhanced the apparent ileal digestibility of dry matter, gross energy, and crude protein. Indeed, SMBS shows considerable promise in detoxifying DON; however, it is essential to emphasize that workers must wear appropriate protective gear when handling SMBS, as it releases sulfur dioxide (SO_2_) under hydrothermal conditions, such as during the pelleting process. In the swine industry, several SMBS-based commercial products have shown significant potential for detoxifying DON in pigs exposed to diets contaminated with 1–3 mg/kg of DON. Defusion and Defusion Plus products, developed by Cargill Animal Nutrition, consist of a formulation containing 92% SMBS, along with organic acids, fermentation products, and supplementary vitamins and amino acids. Several studies have demonstrated that the inclusion of 2.5 and 5 g/kg Defusion and 1.5 and 5 g/kg Defusion Plus in diets contaminated with 3 mg/kg and 1.1–1.5 mg/kg DON in pigs can mitigate the growth performance deficits induced by DON [36,295]. Moreover, a product developed by NutriQuest that contains 92% SMBS, bentonite, and mineral oil has also demonstrated positive effects on the detoxification of DON in pigs. Shawk et al. [295] outlined the addition of 2.5 and 5 g/kg NutriQuest product to diets contaminated with 1.1–1.5 mg/kg DON in piglets for 35 days could improve growth performance. Overall, SMBS and SMBS-based products demonstrate significant potential for mitigating the adverse effects of DON in pigs; however, further trials are necessary to validate these findings.

#### 5.4.3. DON Levels Between 3 Mg/Kg and 5 Mg/Kg in Diets

Moderately high doses of DON, ranging from 3.0 to 5.0 mg/kg in pigs, have been associated with growth retardation, anorexia, metabolic disorder, diarrhea, and gastrointestinal inflammation [31,342]. Similarly to the approach taken for low doses of DON in pigs, various nutritional intervention strategies have been investigated to mitigate the adverse effects of DON.

Only a limited number of microorganisms have been studied for their ability to detoxify DON at this dosage in pigs. *Lactobacillus rhamnosus* GG, one of the most widely used probiotics, has demonstrated anti-inflammatory effects and is effective in preventing or treating various diseases, including diarrhea and atopic dermatitis, while also preventing intestinal epithelial damage and apoptosis, and maintaining barrier function [343]. Bai et al. [344] demonstrated that the addition of 1.77 × 10^11^ CFU/kg *Lactobacillus rhamnosus* GG to diets contaminated with 3.11 mg/kg DON in pigs for 21 days improved growth performance, and mitigated DON-induced intestinal inflammation and damage. Moreover, the freeze-dried powder of *Lactobacillus rhamnosus* GG also exhibited a protective effect against DON-induced toxicity in the kidneys of pigs [345]. *Bacillus subtilis* strain ASAG 216, isolated from the intestine of a donkey, demonstrated the ability to detoxify 81.1% of DON within 8 h under broad temperature (35–50 °C) and pH (6.5–9.0) conditions in vitro [257]. Subsequently, supplementation 1 × 10^8^ CFU/mL *Bacillus subtilis* strain ASAG 216 via drinking water to pigs fed diets contaminated with 3.6 mg/kg DON for 42 days effectively improved growth performance, alleviated DON-induced oxidative stress, attenuated intestinal inflammation, and impaired intestinal barrier [346]. The DON detoxification mechanism of *Bacillus subtilis* strain ASAG 216 in pigs involves the biotransformation of DON into DOM-1, as evidenced by the measurement of DON and DOM-1 concentrations in the serum, liver, and kidneys. Among all in vivo experiments on microbial detoxification of DON, *Bacillus subtilis* strain ASAG 216 was the only one demonstrated to convert DON to DOM-1 in vivo, and it did so under conditions involving a relatively high dietary level of DON. For microbial detoxification of DON in pigs, challenges remain regarding microbial safety, stability, and a lack of extensive animal trials to confirm effectiveness, requiring further research to identify suitable candidates.

Similarly to microorganisms, only a limited number of mycotoxin binders have been investigated for their ability to detoxify DON at this dosage in pigs. Because of DON’s inherent physical and chemical stability, a single mycotoxin binder cannot fully neutralize it. Therefore, mycotoxin binders are often combined with plant extracts, organic acids, and antioxidants. However, the results of these combinations in detoxifying DON in pigs have been inconsistent. Several studies have found that these combinations were ineffective in detoxifying DON in pigs. For instance, Mycosorb™, a polymeric glucomannan mycotoxin adsorbent derived from *Saccharomyces cerevisiae* and developed by Alltech Ireland (Dunboyne, County Meath, Ireland), was evaluated in piglets. The inclusion of 2 g/kg Mycosorb™ in diets contaminated with 4.4 mg/kg DON, administered for 35 days, did not improve growth performance and was ineffective in reducing DON absorption [347]. Similarly, Integral, a product developed by Alltech Inc. that contains yeast, was evaluated in growing-finishing pigs. The supplementation of 1–2 g/kg Integral in diets contaminated with 4 mg/kg DON, fed for 115 days, did not enhance growth performance [348]. Moreover, NutraMix, a product developed by Canadian Bio-Systems Inc. (Calgary, AB, Canada), containing immune-modulating components such as vitamins, a yeast product (dehydrated yeast autolysate), and an inorganic adsorbent (montmorillonite clay), was also assessed in piglets. The addition of 2 g/kg NutraMix to diets contaminated with 1.4 and 3.5 mg/kg DON, fed for 42 days, failed to improve growth performance or alleviate DON-induced intestinal morphological damage, even at the lower contamination level of 1.4 mg/kg. However, certain combinations have demonstrated positive effects in detoxifying DON in pigs. For instance, three yeast-based mycotoxin binders were evaluated in diets contaminated with 3.2 mg/kg DON in piglets for 34 days, revealing that these binders, which incorporated functional components such as clay, inactivated yeast, botanicals, and antioxidants, could improve growth performance while potentially enhancing immune function, gut health, and reducing oxidative stress [349]. Similar results were also obtained by Weaver et al. [350]. Furthermore, UNIKEPlus, developed by Adisseo (Paris, France), consists of adsorbent clay minerals, inactivated fermentation extracts of *Saccharomyces cerevisiae*, and blends of antioxidants, preservatives, and botanicals, and were evaluated in piglets. The addition of 1 and 5 g/kg UNIKEPlus to diets contaminated with 4.5 mg/kg DON for 67 days effectively mitigated the adverse impacts on growth performance and oxidative stress [351]. These varying findings suggest that these products may not effectively detoxify DON under different conditions, highlighting the need for additional and diverse animal trials to further validate these results. Furthermore, the differences in the composition of individual products may also contribute to these discrepancies.

Compared to microorganisms and mycotoxin binders, a larger number of studies have examined a wider range of nutritional interventions at this dosage to mitigate the adverse effects of DON in pigs, including plant extracts, antimicrobial peptides, vitamins, short-chain fatty acids, and their combinations. Plant extracts such as resveratrol, baicalin, berberine, and quercetin have showed potential in mitigating the adverse impacts of DON in pigs when dietary DON levels range from 3.0 to 5.0 mg/kg. The addition of 0.3 g/kg resveratrol in diets contaminated with 3.8 mg/kg DON in piglets for 28 days improved gut health during DON challenge by enhancing intestinal barrier function, reducing intestinal inflammation and oxidative damage, and positively modulating the gut microbiota [352,353,354]. The inclusion of 0.04 g/kg Chinese medicine berberine in diets contaminated with 4 mg/kg DON for piglets over 21 days enhanced growth performance, increased antioxidant enzyme activity, reduced the expression of pro-inflammatory cytokine genes, and elevated the expression of tight junction protein genes in the small intestine [355]. Additionally, another Chinese medicine baicalin has also demonstrated potential in alleviating the negative effects of DON in pigs. Liao et al. [356] reported that the supplementation of 1 g/kg baicalin to diets contaminated with 4 mg/kg DON in piglets for 14 days could improve growth performance, alleviate intestinal inflammatory and oxidative damage. Similar findings were reported by Zha et al. [357,358], who illustrated that the addition of 5 g/kg baicalin–copper or 5 g/kg baicalin–zinc complexes to diets contaminated with 4 mg/kg DON for 14 days in piglets could restore growth performance, mitigate the inflammatory response, and modulate the gut microbiota. Finally, a recent study by Liu et al. [359] demonstrated that the addition of 0.1 g/kg quercetin (a type of polyhydroxy flavonoid) to diets contaminated with 4 mg/kg DON in piglets for 21 days could restore growth performance and ameliorate DON-induced intestinal injury and barrier dysfunction. Antimicrobial peptides were also effective in decreasing the adverse effects of DON in pigs. Xiao et al. [360,361] outlined that the addition of 4 g/kg composite antimicrobial peptides to diets contaminated with 4 mg/kg DON in piglets for 30 days did not enhance growth performance but improved feed efficiency, immune function, antioxidant capacity, alleviated organ damage, and enhanced intestinal morphology. Vitamins, such as vitamin D, exhibit immunoregulatory functions that can reduce the inflammatory response in pigs, suggesting that the inclusion of vitamin D in the diets of pigs may offer protective benefits against inflammation and oxidative stress induced by DON [362]. In a recent study, Sauvé et al. [363] found that the inclusion of 200 and 2200 IU/kg vitamin D3, as well as 2000 IU/kg 25-hydroxyvitamin D3, in diets contaminated with 4.9 mg/kg DON in piglets for 21 days did not improve growth performance, but it enhanced the immune response. Similar findings were received by Frankič et al. [364], who demonstrated that supplementation with 0.1 g/kg vitamin E did not improve production parameters affected by 4 mg/kg DON-contaminated diets in piglets for 14 days. Also, Van Le Thanh et al. [365] reported that while vitamin complexes or combinations of vitamins with antioxidant feed additives did not restore growth performance, they were able to partially alleviate oxidative stress in pigs. Sodium butyrate has been shown to mediate various biological processes, including cholesterol metabolism, possibly through the regulation of histone acetylation, and is believed to mitigate the adverse effects of DON in pigs [366]. The addition of 2 g/kg sodium butyrate to diets contaminated with 4 mg/kg DON in pigs for 28 days improved growth performance and alleviated intestinal barrier dysfunction [367]. Indeed, certain nutritional interventions could partially mitigate the adverse impacts of DON in pigs, but their cost and safety must be carefully evaluated.

Consistent with its effectiveness at DON levels of 1 to 3 mg/kg, the Defusion product also demonstrated positive results at DON concentrations of 3 to 5 mg/kg in pigs [36,348,368,369]. Interestingly, a previous study by Bouchard et al. [370] found that the addition of 3 g/kg Defusion to diets contaminated with 4.49 mg/kg DON in pigs reduced intestinal absorption of DON but also decreased the apparent ileal digestibility of dry matter, energy, acid detergent fiber, ether extract, and phosphorus. This finding raises an important question regarding whether the individual components of Defusion product should be adjusted to ensure effective reduction in intestinal DON absorption without compromising the absorption of other nutrients in pigs. As previously noted, the direct addition of SMBS to feed requires workers to wear protective eyewear to prevent its reaction with water during the pelleting process, which can produce sulfur dioxide (SO_2_) and pose health risks. Additionally, direct incorporation of SMBS may reduce the nutrient content of the feed. Hence, encapsulating SMBS for targeted release at specific sites presents promising application potential. In a recent study, Yu et al. [371] employed a pH-sensitive polymer, Eudragit L100-55, to encapsulate SMBS using a fluidized bed coating technique, demonstrating that the inclusion of 4 g/kg SMBS-encapsulated Eudragit L100-55 microparticles in diets contaminated with 3.3 mg/kg DON in piglets for 28 days improved growth performance, preserved intestinal morphology, attenuated the inflammatory response, and enhanced intestinal barrier function. These findings further indicate that SMBS is highly effective in detoxifying DON in pigs, even at levels ranging from 3 to 5 mg/kg and holds significant potential for industrial application.

#### 5.4.4. DON Levels Exceeding 5.0 Mg/Kg in Diets

Previous studies have demonstrated that high concentrations of DON (≥5 mg/kg) in pigs can cause emesis and feed refusal, along with severe symptoms such as hemorrhagic diarrhea, circulatory shock, and, in some cases, death [31,44,47,372,373]. To address this issue, two approaches are commonly used: the first combines physical, chemical, and biological detoxification strategies with subsequent in vivo nutritional strategies, while the second relies directly on in vivo nutritional strategies. Binders or adsorbents used in mycotoxin detoxification play a crucial role in animal and food safety. These substances work by binding to mycotoxins in the gastrointestinal tract, preventing their absorption into the bloodstream and subsequent toxic effects. Several studies have found that the addition of binders or adsorbents, including aluminosilicate, algae-modified montmorillonite clay, Mycofix^®^ Plus (Biomin, Inzersdorf-Getzersdorf, Austria) containing adsorbing and enzymatic components, bentonite, and yeast cell wall, failed to counteract the detrimental effects of DON in pigs [46,323,374,375,376]. The results indicated that the inherent physical and chemical stability of DON might limit its ability to be absorbed in the intestine by binders or adsorbents, particularly when presented at high levels in diets. Moreover, the inclusion of relatively large amounts of binders or adsorbents in diets might result in non-specific binding of nutrients and micronutrients, potentially impairing their digestion and absorption [374]. Interestingly, the glucomannan extracted from the yeast cell wall demonstrated partial effectiveness in mitigating the adverse impacts of DON in pigs. Swamy et al. [377] reported that supplementing 0.05%, 0.1%, and 0.2% glucomannan to diets contaminated with 5.5 mg/kg DON in pigs for 21 days did not enhance growth performance but effectively prevented mycotoxin-induced alterations in neurochemistry and serum Ig concentrations. Similarly, Diaz-Llano and Smith [378] found that adding 0.2% glucomannan to diets contaminated with 5.5 mg/kg of DON in gilts increased the percentage of pigs born alive compared to gilts fed DON-contaminated diets without the supplement. Like binders or adsorbents, biodegradation in pigs may not completely detoxify DON when present at elevated levels in diets. Sayyari et al. [226] illustrated that the addition of 1 g/kg *Coriobacteriacea* strain DSM 11,798 to 5.7 mg/kg DON-contaminated diets in weaning piglets for 42 days was ineffective in biotransformation DON into less toxic components and in preventing mycotoxin-related effects. Remarkably, the addition of 0.5% of a mixture containing enzymes, microorganisms, minerals, and plant extracts to the diets of pigs for 12 days partially alleviated the negative effects of dietary DON on the gain-to-feed ratio [46]. Nutrients, such as amino acids and vitamins, have been shown to effectively alleviate intestinal toxicity induced by DON through indirect mechanisms. In particular, Wu et al. [379] showcased that supplementing diets contaminated with 6 mg/kg DON with 0.5% arginine and 0.5% glutamine for 28 days in growing pigs effectively improved the impairments induced by DON stress, including modulation of immune-relevant cytokines. Wu et al. [380] also found that adding 1.0% arginine to 6 mg/kg DON-contaminated diets in weaning piglets for 28 days could alleviate the intestinal impairment caused by DON challenge. Moreover, Sauvé et al. [363] reported that supplementation with vitamin E, C, and 25-hydroxyvitamin D3 in pigs fed diets contaminated with 5.1 mg/kg of DON for 21 days did not enhance grower performance; however, vitamin E and C supplementation reduced the circulating and hepatic oxidative stress. Collectively, these studies indicate that complete detoxification of DON in vivo may not be achievable when its concentration exceeds 5.0 mg/kg. Consequently, it is crucial to implement in vitro detoxification methods first, followed by additional in vivo detoxification through nutritional strategies once DON levels have been reduced to an acceptable threshold.

## 6. The Need for Generative Artificial Intelligence (AI) in DON Detoxification

While enzymatic degradation of DON shows great potential, its practical application faces several challenges, including enzyme identification, specificity, stability, cost, integration into existing systems, and regulatory hurdles. Addressing these limitations is essential for advancing the use of enzymatic methods for DON detoxification. Among these challenges, identifying new mycotoxin-degrading enzymes, particularly for DON, is of paramount importance. Recent research has increasingly focused on alternative approaches to enzyme identification, such as leveraging artificial intelligence (AI) and machine learning algorithms. For instance, the number of protein sequences in Uniprot (https://uniprot.org/) has surpassed 109 million, providing a vast resource for screening potential DON-detoxifying enzymes.

The identification of detoxifying enzymes is crucial for various applications, including environmental cleanup, drug development, and food safety. This identification is crucial for the mycotoxin contamination mitigation. However, traditional methods of enzyme identification are labor-intensive and economically constrained, with additional concerns about the potential toxicity of degradation products [381]. This is where AI, particularly machine learning and deep learning algorithms, becomes invaluable. These technologies can analyze extensive datasets to uncover patterns and relationships that are not easily discernible by human researchers. AI algorithms can evaluate genetic sequences, protein structures, and biochemical properties to predict which enzymes might possess detoxifying capabilities. They enable the search for bacterial and fungal strains with detoxifying enzymes, the investigation of the cellular machinery of these strains, and the analysis of enzyme molecular structures. Additionally, AI can integrate and analyze data from diverse sources—such as genomic databases, experimental results, and scientific literature, providing a comprehensive view that enhances the accurate identification of target enzymes [382].

By automating and accelerating the analysis process, AI not only improves the efficiency and accuracy of enzyme identification but also facilitates the discovery of novel enzymes that might be overlooked by conventional methods. This capability is particularly crucial for addressing urgent environmental issues, such as the degradation of pollutants and toxic substances, where both speed and precision are vital and are increasingly important for identifying mycotoxins degrading enzymes. For instance, Zhang et al. [381,382] demonstrated the effectiveness of an AI framework that integrated data-driven prediction models with a rapid cell-free protein expression (CFPE) system in mycotoxin degrading enzymes search. Their approach, known as positive unlabeled learning-based enzyme promiscuity prediction (PU-EPP), successfully identified 15 new enzymes with significant degradation activity for mycotoxins OTA and ZEA within less than 30 days. They were also able to pinpoint critical enzyme residues based solely on sequence-level information. This example highlights the potential of similar AI-driven frameworks—whether incorporating multi-omics approaches or not—for discovering enzymes capable of degrading DON. The use of PU-EPP to explore enzymes for DON warrants further investigation.

Compared to traditional methods such as microbial screening and functional metagenomics, data-driven approaches offer the advantage of screening large-scale enzyme-substrate libraries more efficiently and cost-effectively [383]. In terms of structural bioinformatics, molecular docking software such as EnzyDOCK (CHARMM program), AutoDock suite (https://autodocksuite.scripps.edu (accessed on 10 July 2025), AutoDock4), VINA (https://vina.scripps.edu (accessed on 10 July 2025), AutoDock Vina 1.1.2), Genetic Optimization for Ligand Docking (GOLD, CCDC/Astex validation set), and FRED (OEDocking 3.0) can be incorporated into AI-based frameworks to enhance the search for DON-degrading enzymes [384,385,386].

Predicting the efficacy of enzymes in degrading DON is crucial for ensuring food safety, protecting animal health, and promoting economic and environmental sustainability. Accurate predictions enable the development of effective, cost-efficient, and eco-friendly solutions for managing DON contamination. Machine learning models use mathematical and statistical functions to analyze existing datasets and predict enzyme performance on new data. Publicly available datasets such as UniProt, BindingDB, BRENDA, and RxnFinder provide valuable information on interactions, binding affinities, and Michaelis constants between proteins and compounds, including DON [383]. Machine learning has significantly enhanced our ability to predict enzyme properties, including activity, stability, and specificity. By leveraging extensive datasets of enzyme sequences and structural information, these models can forecast how modifications in enzyme design will affect performance. For example, tools like EZYDeep enable enzyme function predictions based on sequence information [387]. Additionally, GotEnzymes, available at https://metabolicatlas.org/gotenzymes (accessed on 10 July 2025), offers an interactive web platform for high-throughput enzyme property predictions using AI approaches [388]. Another valuable tool, ALDELE, predicts biocatalytic activity by integrating structural and sequence representations of proteins, along with ligand subgraph representations and overall physicochemical properties [389]. These advancements in machine learning and computational tools are instrumental in accelerating the discovery and optimization of enzymes for DON degradation.

Machine learning algorithms play a crucial role in discovering novel enzymes by identifying promising sequences from extensive protein databases and optimizing enzyme properties such as substrate affinity and catalytic efficiency [390]. Numerous studies demonstrate the effectiveness of this approach. For instance, Visani et al. [391] developed a hierarchical multi-label neural network (EPP-HMCNF) to predict which enzymes may interact with specific substrates. Similarly, Mou et al. [392] integrated experimental enzyme activity data with extracted protein and ligand features to create predictive models for enzyme substrate scope. These examples highlight the potential of machine learning to enhance enzyme discovery and optimization processes.

Degradation pathways often involve complex multistep reactions to achieve complete toxin degradation. However, AI can expedite the identification of effective single-step reactions. For example, Zhang et al. [381,382] used AI to identify enzymes capable of degrading OTA and ZEA through optimal single-step reactions, resulting in products with lower toxicity. This AI framework can be combined with pathway design tools, such as novePathFinder, to determine more comprehensive degradation pathways for mycotoxins [393]. Additionally, AI-driven approaches have the advantage of continuously learning and improving from new data, leading to increasingly refined and effective identification strategies. Therefore, integrating AI into enzyme identification represents a significant advancement, expanding the possibilities in biochemical research and its practical applications [382,383].

### Challenges and Future Directions

Degrading mycotoxins often requires multiple enzymatic reactions to produce by-products with significantly reduced toxicity. In many cases, a single enzyme is insufficient for complete degradation, necessitating a series of enzymatic steps. For example, Carere et al. [260,265] described a two-component enzymatic pathway in the epimerization of DON by *Devosia* mutans strain 17-2-E-8. This pathway involves two enzymes, DepA and DepB. DepA first oxidizes DON to 3-keto-DON, and then DepB reduces 3-keto-DON to 3-epi-DON, substantially decreasing toxicity. Understanding all the enzymes involved in complete degradation is crucial. Often, this requires identifying multiple enzymes and degradation pathways for a specific mycotoxin.

Traditional enzyme-mining methods can make this process challenging and time-consuming. However, AI can streamline and accelerate the identification of these complex enzymatic systems. Despite its advantages, AI faces several challenges and limitations, such as data scarcity. Effective AI models depend on large, diverse datasets. For DON-degrading enzymes, high-quality data on enzyme properties, interactions with DON, and experimental outcomes may be sparse. Additionally, variability in experimental conditions, methodologies, and reporting standards can lead to inconsistent data, complicating the training of robust AI models that generalize well across different datasets.

The complexity of biological systems presents a significant challenge for AI-driven enzyme mining. Enzymatic activity is influenced by various factors such as pH, temperature, and enzyme structure, which can introduce considerable biological variability. AI models may struggle to account for these factors and accurately predict enzyme efficacy under diverse conditions. Additionally, the interactions between DON and potential degrading enzymes are complex and not always fully understood, which means AI models might fail to capture all relevant biological interactions or predict the effects of enzyme modifications.

Moreover, AI models, including those based on machine learning and deep learning, are limited by the accuracy of their underlying algorithms. If the algorithms are not well-suited to the specific challenges of enzyme degradation, the predictions may be unreliable. There is also a risk of overfitting, particularly if the dataset is small or not representative of the full spectrum of enzyme variants. Overfitted models may perform well on training data but fail to generalize to new, unseen data. These limitations underscore the need for robust and well-calibrated AI models to improve predictions and outcomes in enzyme mining.

To achieve effective enzyme optimization, a combination of machine learning and high-throughput laboratory experiments will be increasingly essential. Integrating AI with microbial and enzymatic strategies, alongside physical or chemical methods, could significantly enhance the overall efficacy of DON detoxification. Moreover, incorporating multi-omics approaches with AI can further refine enzyme optimization.

Multi-omics strategies, which integrate genomics, proteomics, transcriptomics, and metabolomics data, combined with machine learning algorithms, provide a comprehensive view of the biological systems involved in DON degradation and other mycotoxins [258,390]. AI algorithms can analyze complex multi-omics data to uncover patterns and correlations that traditional enzyme-mining methods may overlook. These insights can elucidate how factors like protein modifications and metabolic pathways influence enzyme function. By leveraging this information, AI can optimize enzyme properties such as specificity and efficiency for more effective DON degradation.

## 7. Conclusions

Contaminated DON food and feed has become a problem that must be urgently solved. This review paper systematically illustrates the absorption, metabolism of DON in pigs and current strategies and techniques for detoxification of DON in feed. Although numbers of DON reducing methods have been investigated, we discovered that these approaches cannot completely eliminate DON contamination in cereals or remove the adverse effects on animals or human health in vitro. Additionally, even long-term exposure to lower than regulation concentrations of DON also showed negative effects on pigs. Therefore, to minimize the impact of DON, some in vivo approaches such as nutritional interventions and the use of microorganisms and enzymes should be deeply researched in the future. Moreover, the mechanisms of these in vivo methods also need to be identified in the future. Mycotoxins degrading enzymes mining is currently heavily dependent on costly and time consuming in vitro and in vivo experiments. AI techniques are increasingly being used to identify and characterize enzymes that degrade DON due to its ability to analyze vast amounts of data and predict enzyme functions in a time and cost-effective manner.

## Figures and Tables

**Table 1:** The impacts of different levels and sources of deoxynivalenol (DON) on swine.

Stage	Level of DON in Diet	Source	Effects	Reference
Weaning	2.88 mg/kg	Naturally contaminated wheats	Reduced BW	[34]
Weaning	3.5 mg/kg	Naturally contaminated wheats	Reduced ADG, impaired intestinal barrier and immunological functions	[35]
Weaning	3.0, 4.0 mg/kg	Naturally contaminated corn DDGS	Decreased ADG, ADFI, and BW	[36]
Weaning	3.3–3.8 mg/kg	Naturally contaminated wheats	Decreased BW	[37]
Weaning	5.5 mg/kg	Naturally contaminated corns	Decreased ADG and ADFI	[38]
Weaning	2.0 mg/kg	Naturally contaminated corn DDGS	Decreased ADG, ADFI, and G/F	[39]
Weaning	1.8 mg/kg	Naturally contaminated corn DDGS	Decreased G/F	[40]
Growing	1.0 and 3.0 mg/kg	Naturally contaminated corns	Reduced feed intake and weight gain	[41]
Growing	4.0 mg/kg	Naturally contaminated corns	Decreased feed intake and lose weight	[42]
Growing	0.6, 1.8, 4.7 mg/kg	Naturally contaminated oats	Decreased feed intake	[43]
Growing	3.0, 6.0, and 12.0 mg/kg	Naturally mouldy corns	Weight loss, live injury, oxidation stress, and malabsorption of nutrients	[44]
Growing	0.9 mg/kg	Commercial	Decreased weight gain and induced histomorphological alterations	[45]
Growing	6.89 mg/kg	Naturally contaminated barley	Reduced ADFI and ADG	[46]
Growing-Finishing	5.65–6.15 mg/kg	Naturally contaminated wheats	Reduced feed consumption and live weight gain	[47]
Growing-Finishing	1.0, 3.0, and 5.0 mg/kg	Naturally contaminated wheats	Reduced growth performance	[48]
Finishing	3.02 mg/kg	Naturally contaminated corns	Decreased ADG and ADFI	[49]
Finishing	1.0, 3.0, and 5.0 mg/kg	Naturally contaminated wheats	Decreased BW	[50]

Note: BW, body weight; ADG, average daily gain; ADFI, average daily feed intake; G/F ratio, gain to feed ratio.

**Table 2:** A summary of physical strategies for deoxynivalenol (DON) detoxification.

Methods	Used Measures and Reagents	Detoxification Efficiency	Reference
Sorting	Multiple high speed optical sorting	The 0.6–20 mg/kg DON in the sorted wheat was reduced to 51% of its original level using one-pass sorting.	[132]
Sorting	Gravity separation	A reduction of 30.85% in 899.1–2442.4 µg/kg DON in unseparated wheat was found.	[133]
Washing	Water and sodium carbonate solution	A single water rinsing step decreased 40.3 mg/kg DON and 0.94 mg/kg ZEA concentrations by 44% in corn, and further reduction by 35% was achieved through additional soaking in a 0.1 mol/L aqueous sodium carbonate solution.	[134]
Dehulling	Gentle debranning technology	Gentle debranning technology resulted in the removal of 80.01% of 4.66 Log CFU/g microorganisms and 15.82% of 6.88 µg/kg DON.	[135]
Heating	Baking	Baking maize samples containing 3905–4286 ng/g DON at 240 °C for 15 min, 200 °C for 30 min, and 150 °C for 180 min resulted in reductions of 31.8%, 35.7%, and 37.3%, respectively.	[136]
Heating	Cooking, roasting, and extrusion cooking	Cooking reduced to 428–875 µg/kg DON and 59–182 µg/kg ZEA in grain samples by 8% and 11% at 200 °C for 30 min, whereas roasting and extrusion cooking at the same condition achieved more substantial reductions of 40% for DON and 46% for ZEA and 75% for DON and 80% for ZEA, respectively.	[137]
Heating	Baking	The degradation of DON ranged from 0 to 21% in crackers (691–1429 µg/kg DON), 4 to 16% in biscuits (872–1036 µg/kg DON), and 2 to 5% in bread (1114–1435 µg/kg DON) under varied processing conditions.	[138]
Heating	Steam-softening	Steam-softening led to the reduction in DON, DON-3-G, and total DON in flakes, retaining 41%, 60%, and 46%, respectively.	[139]
Milling	Industrial roller-grinder	The industrial roller-grinder yielded a reduction of 77.8% to 80.5% in 1400–1900 µg/kg DON after milling the wheat grain.	[140]
Milling	Dry milling	A 53% decrease in 294 µg/kg DON was observed in wheat germ following the dry milling of cleaned whole grain.	[141]
Milling	Roller milling and precision milling	The roller mill reduced 14.3–38.9 µg/g DON by 36.7% from the kernel, while the precision mill decreased 14.3–38.9 µg/g DON by 85.1% from the dehulled kernel.	[142]
Milling	Laboratory mill machine	Milling yields a reduction of 58.02% in total bacteria and 32.62% in 899.1–2442.4 µg/kg DON in wheat.	[133]
Extrusion	Twin screw extruder	At temperatures of 140, 160, and 180 °C, each with a moisture content of 15%, there was a reduction in 1000 µg/kg DON by 18.9%, 23.4%, and 20.5%, respectively, in wheat.	[143]
Irradiation	Gamma-ray irradiation	The 103–287 µg/kg of DON in wheat was reduced by 41% following 2 kGy gamma-ray irradiation.	[144]
Light	Ultraviolet irradiation	In dry grain samples, the UV treatment (24 mW/cm^2^ at 254 nm UV-C) completely eliminated 30 mg/kg of ZEA in 15 min and 30 mg/kg of DON in 20 min.	[145]
Light	Intense pulsed light	The application of intense pulsed light treatment resulted in a significant reduction of 35.5% in 1.45 mg/kg DON within barley samples following the administration of 180 pulses within 1 min.	[146]
Cold atmospheric plasma	Dielectric barriers discharge atmospheric pressure cold plasma	Dielectric barriers discharge atmospheric pressure cold plasma, applied at 30 kV, led to a 100% reduction of 37.0 µg/mL DON solution within 5 min, surpassing the 75.9% reduction observed in dry conditions in 60 min.	[147]
Ultrasound	Pulsed ultrasound	Applying ultrasound at power intensity of 4.4 W/cm^3^ and a duty cycle of 66.7% led to a 43.2% decrease in the content of 250–4000 µg/L DON.	[148]

**Table 3:** A summary of chemical strategies for deoxynivalenol (DON) detoxification.

Methods	Products	DON Concentration	Assay Conditions	Efficiency	Reference
Ammonization, oxidation, and reduction	Wheat	1000 mg/kg	30% chlorine for 0.5 h, 100% ammonia carbonate at 132 °C for 18 h, and 1% sodium bisulfite solution for 6 days	100%, 85%, and 100%	[194]
Ammonization	Wheat kernels	11.3 μg/kg	4.8% ammonia vapours at 115 °C for 2 h	72.4%	[195]
Reduction	Corn	7.2 mg/kg	Autoclave at 121 °C for 1 h with 8.33% aqueous sodium bisulfite	95%	[196]
Reduction	Wheat	7.6 mg/kg	10 g/kg of sodium metabisulfite at 100 °C with 22% moisture for 15 min	96.3%	[197]
Reduction	Barley	1.4 mg/kg	200 g/L of sodium bisulfite solutions under soaking for 0.5 h	92.9%	[198]
Reduction	Triticale kernels	6.63 mg/kg	5 g/kg of sodium metabisulfite and 10 g/kg of propionic acid at 15% moisture for 63 days	96%	[199]
Reduction and alkaline hydrolysis	Maize	43.4 mg/kg	5 g/kg of sodium metabisulfite, 10 g/kg of methylamine, and 20 g/kg of calcium hydroxide at 20% moisture for 30 min at 80 °C	91%	[200]
Reduction	Maize kernels and maize meal	51.6 mg/kg	10 g/kg of sodium bisulfite and 30% moisture for 79 days	100%	[201]
Reduction	Maize	63.93 mg/kg	5 g/kg of sodium metabisulfite and 15 g/kg propionic acid for 9 weeks	85.6%	[202]
Reduction	Maize	5.36 mg/kg	5 g/kg of sodium metabisulfite and 15 g/kg propionic acid with 20% moisture for 79 days	84.5%	[203]
Alkaline hydrolysis	Barley	16.1 mg/kg	1 mol/L sodium carbonate solution at 22 °C for 30 min	72–74%	[152]
Alkaline hydrolysis	Barley	18.4 mg/kg	1 mol/L sodium carbonate solution at 80 °C for 8 days	100%	[204]
Ozone	Wheat	1000 mg/kg	Moist ozone (1.1 mol %) and dry ozone (1.1 mol %) for 1 h	90% and 70%	[194]
Ozone	DON solution wheat	1 μg/mL; 10 mg/kg	10 mg/L of gaseous ozone for 30 s; 10 mg/L of gaseous ozone with 17% moisture content for 12 h	93.6%; 57.3%	[205]
Ozone	Whole wheat flour	3.89 mg/kg	100 mg/L of ozone level with 20% moisture content for 60 min	78.7%	[206]
Ozone	Whole wheat flour	2748 μg/kg	65 mg/L of gaseous ozone with 25% moisture content for 180 min	80%	[207]
Ozone	Wheat	247–294 μg/kg	55 g/h of ozone for 6 h	44%	[208]
Ozone	DON solution	10.76 mg/L	14.5 mg/L of aqueous ozone at a flow rate of 80 mL/min for 20 min	97.95%	[209]
Ozone	Wheat kernels	1.29 mg/kg	60 mg/L of ozone gas for 2 h	33.33%	[210]
Ozone	Corn and wheat	488–2211 μg/kg	3 mg/L of ozone for 8 h in the lab and 96 h in the bran	40% and 50%	[211]

**Table 4:** A summary of microbes with deoxynivalenol (DON) detoxification.

Microbes	Source	Mechanism	Product	Detoxification Efficiency	Reference
A bacterial strain BBSH797	Bovine rumen	Anaerobic de-epoxidation at C12-C13	DOM-1	-	[225]
*Coriobacteriaceum* DSM 11798	Bovine rumen	Anaerobic de-epoxidation at C12-C13	DOM-1	-	[226]
Microbioal culture C133	Fish digesta	Anaerobic de-epoxidation at C12-C13	DOM-1	Culture C133 converted 50 µg/mL of DON to DOM-1 in full growth medium for 96 h at 15 °C.	[227]
*Bacillus* sp. LS100	Chicken digesta	Anaerobic de-epoxidation at C12-C13	DOM-1	Under anerobic conditions for 24 h at 37 °C, the *Bacillus* sp. LS100 completely transformed 1000 mg/mL of DON into DOM-1.	[228]
*Clostridium* sp. WJ06	Chicken intestines	Anaerobic de-epoxidation at C12-C13	DOM-1	Twenty mg/kg of DON can undergo transformation into DOM-1 with a degradation rate over 90% by WJ06.	[229]
*Eggerthella* sp. DII-9	Chicken intestines	Anaerobic de-epoxidation at C12-C13	DOM-1	Bacterium DII-9 eliminated 100 µg/mL DON into DOM-1 at 20–45 °C and pH 5–10.	[230]
*Slackia* sp. D-G6	Chicken intestines	Anaerobic de-epoxidation at C12-C13	DOM-1	*Slackia* sp. D-G6 converted 25 µg/mL of DON into DOM-1 at 37–47 °C and pH 6–10.	[231]
A bacterial consortium YM-1	Chickens	Anaerobic de-epoxidation at C12-C13	DOM-1	Under anaerobic conditions for 24 h, 50 μg/L of DON experienced a 99.2% de-epoxidation.	[232]
*Serratia*, *Clostridium*, *Citrobacter*, *Enterococcus*, *Stenotrophomonas*, and *Streptomyces*	Soil	Aerobic de-epoxidation at C12-C13	DOM-1	Under aerobic conditions for 60 h at 12–40 °C and pH 6.0–7.5, the enriched culture completely converted 50 µg/mL DON into DOM-1.	[233]
*A bacterial consortium PGC-3*	Soil	Aerobic de-epoxidation at C12-C13	DOM-1	PGC-3 converted 100 µg/mL DON into DOM-1 under aerobic conditions at 20–37 °C and pH 5–10.	[234]
*Desulfitobacterium* sp. PGC-3-9	Soil	Aerobic and anaerobic de-epoxidation at C12-C13	DOM-1	PGC-3-9 degraded 168.74 μM of DON into DOM-1 ability at 5–10 °C and pH 5–10.	[235]
*Mixed culture D107*	Soil	Aerobic oxidation of C3	3-keto-DON	D107 oxidated 200 µg/mL of DON into 3-keto-DON for 5 days at 20 °C in aerobic conditions.	[236]
*Devosia* insulae A16	Soil	Aerobic oxidation of C3	3-keto-DON	Under neutral pH and at 35 °C, the bacterial strain A16 degraded 88% of 20 mg/L DON into 3-keto-DON within 48 h.	[237]
*Pelagibacterium halotolerans* ANSP101	Ocean	Oxidation of C3	3-keto-DON	The bacterial strain ANSP101 could oxidate 50 μg/mL of DON into 3-keto-DON by 80% at 40 °C and pH 8 for 24 h.	[238]
Bacterial *consortium* C20	Wheat	Aerobic oxidation of C3	3-keto-DON	Under aerobic condition for 5 days at 30 °C and pH 8, the bacterial consortium 20 could highly degrade 70 μg/mL of DON into 3-keto-DON.	[239]
Bacterial *consortium* IFSN-C1	Soil	Oxidation of C3	3-keto-DON	The bacterial consortium IFSN-C1 could degrade 10 μg/mL of DON into 3-keto-DON by 86.5% at pH 8 and 37 °C.	[240]
Recombinant plasmid pPIC9K-QDDH	-	Oxidation of C3	3-keto-DON	Within 12 h, the recombinant QDDH transformed 78.46% of 20 μg/mL DON into 3-keto-DON.	[241]
A synthetic bacterial consortium consisting of *Devosia* sp. A8 and *Paracoccus yeei* A9	Soil	Oxidation of C3	3-keto-DON	The synthetic bacteria A8 and A9 could detoxify 10, 100, and 200 μg/mL of DON into 3-keto-DON within 6, 36, and 84 h by 92.48%, 93.68%, and 77.15%, respectively.	[242]
A bacterial *consortium* consisting *Pseudomonas* sp. SD17-1 and *Devosia* sp. SD17-2	*Tenebrio molitor* larval feces	Oxidation of C3	3-keto-DON	The microbial consortium efficiently oxidized 50 μg/mL of DON to 3-keto-DON within 72 h at 30 °C and a pH range of 8.0–9.0.	[243]
*Ketogulonicigenium vulgare* D3_3	*Tenebrio molitor* larval feces	Anaerobic oxidation of C3	3-keto-DON	The bacterial isolate D3_3 achieved complete oxidation of 50 μg/mL of DON to 3-keto-DON within 12 h at 30 °C and pH 7.0–9.0.	[244]
*Citrobacter freundii*	Rice root-linked soil	De-epoxidation at C12-C13 and oxidation of C3	DOM-1 and 3-keto-DON	Under conditions of pH 7 and 37 °C within 72 h, *C. freundii* exhibited the ability to degrade over 90% of DON.	[245]
*Nocardioides.* WSN05-2	Soil	Epimerization of C3	3-epi-DON	The isolate bacterium WSN05-2 completely eliminated 1000 μg/mL of DON in 10 days.	[246]
*Devosia mutans* 17-2-E-8	Soil	Aerobic epimerization of C3	3-epi-DON	In an aerobic condition at 25–37 °C and neutral pH for 72 h, 100 μg/mL of DON was converted into 3-epi-DON by 95%.	[247]
*Paradevosia shaoguanensis* DDB001	Soil	Epimerization of C3	3-epi-DON	Strain DDB001 showed complete elimination of 200 mg/L of DON in the full growth medium.	[248]
*Acinetobacter*, *Leadbetterella*, and *Gemmata*	Plant and soil	Epimerization of C3	3-epi-DON	The incubation of the mixed culture with wheat samples (7.1 μg/mL DON) revealed almost total conversion of DON to the less toxic 3-epi-DON.	[249]
A mixed culture *Pseudomonas* sp. Y1 and *Lysobacter* sp. S1	Soil	Epimerization of C3	3-epi-DON	The mixed culture Y1 and S1 completely converted 50 μg/mL of DON into 3-epi-DON in 48 h.	[250]
*Nocardioides* sp. ZHH-013	Soil	Epimerization of C3	3-epi-DON	ZZH-013 converted 168.74 μM of DON into 3-epi-DON at 30 °C for 14 days.	[251]
A mixed culture *Pseudomonas* sp. B6-24 and *Devosia* strain A6-243	Soil	Epimerization of C3	3-epi-DON	The mixed culture B6-24 and A6-243 could biotransform 50 μg/mL of DON in 72 h.	[252]
*Candida parapsilosis* *ACCC 20221*	Yeast	Epimerization of C3	3-epi-DON	ACCC 20,221 demonstrated an 86.59% reduction of 20 μg/mL 3-keto-DON to 3-epi-DON in 48 h.	[241]
*Trichoderma* spp.	-	Glycosylation	D3G	In the presence of *Trichoderma* spp., over 90% degradation of 57 μg/g DON was achieved.	[253]
*Clonostachys rosea* ACM941	-	Glycosylation (15-ADON)	15-ADON-G	ACM941 converted 125–500 μg/mL of 15A-DON into 15A-D3G at 25–28 °C for 10 days.	[254]
*Aspergillus (* *NJA-1* *)*	Soil	Unknown	Unknown	After 14 days cultivation, the rate of 40 mg/L DON biotransformation reached 94.4% at 30 °C.	[255]
*Bacillus licheniformis* YB9	Soil	Unknown	Unknown	YB9 could efficiently detoxify 82.67% of 1 mg/L DON at 37 °C for 48 h.	[256]
*Bacillus subtilis* ASAG 216	Donkey intestine	Unknown	Unknown	ASAG 216 could detoxify 81.1% of 100 mg/L of DON at 35–50 °C and pH 6.5–9.0 for 8 h.	[257]
*Bacillus* sp. HN117 and N22,	Soil and wheat	Unknown	M-DOM	HN117 eliminated 29.0% of 1000 mg/L DON in 72 h, while N22 exhibited a notable increase in DON degradation rate from 7.41% to 21.21% within 120 h at 500 mg/L DON.	[258]
*Devosia* sp. D-G15	Soil	Oxidation and epimerization of C3, unknown	3-keto-DON, 3-epi-DON, Unknown compound C	*Devosia* sp. D-G15 could completely detoxify 100 μg/mL of DON at pH 7.0 and 30 °C for 60 h.	[259]

**Table 5:** A summary of enzymes with deoxynivalenol (DON) detoxification.

Enzyme	Biological Origin	Source	Mechanism	Product	Reference
AKR18A1	*Sphingomonas* S3-4	Soil	Oxidation	3-keto-DON	[15]
Dep A	*Devosia mutans* 17-2-E-8	Soil	Oxidation	3-keto-DON	[260]
QDDH	a *Devosia* strain D6-9	Wheat field	Oxidation	3-keto-DON	[261]
DDH	*Pelagibacterium halotolerans* ANSP101	Ocean	Oxidation	3-keto-DON	[262]
Sorbose dehydrogenase	*Ketogulonicigenium vulgare* Y25	-	Oxidation	3-keto-DON	[263]
YoDDH	*Youhaiella tibetensis*	-	Oxidation	3-keto-DON	[264]
Dep B	*Devosia mutans* 17-2-E-8	Soil	Epimerization	3-epi-DON	[265]
AKR13B2 and AKR6D1	a *Devosia* strain D6-9	Wheat field	Epimerization	3-epi-DON	[261]
DepB_Rleg_ (AKR18)	*Rhizobium leguminosarum*	-	Epimerization	3-epi-DON	[266]
SPG	*Gossypium hirsutum* cv. CCRI12	-	Isomerization	3-ADON and 15-ADON	[267]
DLK06_RS13370	*Acinetobacter pittii*	Soil	Acetylation	3-ADON	[268]
A cytochrome P450 system	*Sphingomonas* sp. strain KSM1	Lake water	Hydroxylation	16H-DON	[269]
Fhb7	*Th. elongatum*	-	Glutathionylation	DON-GSH	[270]
Glutathione S-transferase (GST) Fhb7-GST	*Thinopyrum ponticum*	Wheat	Glutathionylation	DON-13-GSH	[271]
UDP-Glycosyltransferases UGT73C5	*Arabidopsis thaliana*	Plants	Glycosylation	D3G	[272]
UDP-Glycosyltransferases UGT12887	*Tricum aestivum*	Wheat	Glycosylation	D3G	[273]
UDP-Glycosyltransferases HvUGT13248	*Hordeum vulgare*	Barley	Glycosylation	D3G	[274]
UDP-Glycosyltransferases TaUGT4	*Tricum aestivum*	Wheat	Glycosylation	D3G	[275]
UDP-Glycosyltransferases Bradi5g03300	*Brachypodium distachyon*	Plants	Glycosylation	D3G	[276]
UDP-Glycosyltransferases Os79 (Os04g0206600)	*Oryza sativa*	Rice	Glycosylation	D3G	[277]
UDP-Glycosyltransferases HvUGT13248	*Hordeum vulgare*	Barley	Glycosylation	D3G	[278]
UDP-Glycosyltransferases Traes_2BS_14CA35D5D	*Tricum aestivum*	Wheat	Glycosylation	D3G	[279]
UDP-Glycosyltransferases TaUGT-2B and TaUGT-3B	*Tricum aestivum*	Wheat	Glycosylation	D3G	[280]
UDP-Glycosyltransferases TaUGT5	*Arabidopsis thaliana*	Chinese spring	Glycosylation	D3G	[281]
UDP-Glycosyltransferases TaUGT6	*Tricum aestivum*	Wheat	Glycosylation	D3G	[282]
UDP-Glycosyltransferases AsUGT1 and AsUGT2	*Avena sativa*	Oat	Glycosylation	D3G	[283]
UDP-Glycosyltransferases UGT13248	*Hordeum vulgare*	Barley	Glycosylation	D3G	[284]
UDP-Glycosyltransferases (CrUGT3, CrUGT6 and CrUGT9)	*Clonostachys rosea*	-	Glycosylation	15A-D3G	[285]
Extracellular enzyme from *Aspergillus niger*	*Aspergillus niger*	Soil	Unknown	Unknown	[286]

## Data Availability

Not applicable.
